# Trans-cVAE-GAN: Transformer-Based cVAE-GAN for High-Fidelity EEG Signal Generation

**DOI:** 10.3390/bioengineering12101028

**Published:** 2025-09-26

**Authors:** Yiduo Yao, Xiao Wang, Xudong Hao, Hongyu Sun, Ruixin Dong, Yansheng Li

**Affiliations:** School of Information and Control Engineering, Qingdao University of Technology, Qingdao 266520, China

**Keywords:** generative modeling, conditional variational autoencoder, generative adversarial network, transformer

## Abstract

Electroencephalography signal generation remains a challenging task due to its non-stationarity, multi-scale oscillations, and strong spatiotemporal coupling. Conventional generative models, including VAEs and GAN variants such as DCGAN, WGAN, and WGAN-GP, often yield blurred waveforms, unstable spectral distributions, or lack semantic controllability, limiting their effectiveness in emotion-related applications. To address these challenges, this research proposes a Transformer-based conditional variational autoencoder–generative adversarial network (Trans-cVAE-GAN) that combines Transformer-driven temporal modeling, label-conditioned latent inference, and adversarial learning. A multi-dimensional structural loss further constrains generation by preserving temporal correlation, frequency-domain consistency, and statistical distribution. Experiments on three SEED-family datasets—SEED, SEED-FRA, and SEED-GER—demonstrate high similarity to real EEG, with representative mean ± SD correlations of Pearson ≈ 0.84 ± 0.08/0.74 ± 0.12/0.84 ± 0.07 and Spearman ≈ 0.82 ± 0.07/0.72 ± 0.12/0.83 ± 0.08, together with low spectral divergence (KL ≈ 0.39 ± 0.15/0.41 ± 0.20/0.37 ± 0.18). Comparative analyses show consistent gains over classical GAN baselines, while ablations verify the indispensable roles of the Transformer encoder, label conditioning, and cVAE module. In downstream emotion recognition, augmentation with generated EEG raises accuracy from 86.9% to 91.8% on SEED (with analogous gains on SEED-FRA and SEED-GER), underscoring enhanced generalization and robustness. These results confirm that the proposed approach simultaneously ensures fidelity, stability, and controllability across cohorts, offering a scalable solution for affective computing and brain–computer interface applications.

## 1. Introduction

With the continuous advancement of affective computing and human–computer interaction research, electroencephalography (EEG), as a non-invasive neurophysiological measurement technique with high temporal resolution, has shown broad application prospects in fields such as emotion recognition [1,2,3] and cognitive impairment assessment [4,5]. EEG signals reflect the electrophysiological processes of neuronal population activity in the brain and are characterized by strong nonlinearity, non-stationarity, and significant inter-individual variability, making the acquisition and modeling of high-quality EEG data a persistent challenge.

In practice, high-quality EEG data collection not only depends on expensive instrumentation and stringent experimental conditions but is also subject to participant compliance and ethical constraints. As a result, data acquisition is costly, sample sizes are often limited, and the applicability and scalability of deep learning models for EEG analysis are severely restricted [6]. Consequently, developing high-quality and semantically controllable EEG data generation methods has become a focal point of recent research.

Existing studies have attempted to apply generative adversarial networks (GANs) [7,8,9], variational autoencoders (VAEs) [10,11], deep convolutional GANs (DCGANs) [12,13], Wasserstein GANs (WGAN) [14,15], and Wasserstein GANs with gradient penalty (WGAN-GP) [16] to EEG signal generation. However, the following bottlenecks remain:

(1) Traditional VAE architectures can model latent distributions but often produce blurry samples with structural distortion;
(2) Classical GANs have limited temporal modeling capability, making it difficult to capture the dynamic features and spectral structures of EEG signals;
(3) Most existing models lack effective emotion label conditioning mechanisms, hindering their ability to generate EEG data under specific semantic guidance and thereby limiting their usability in practical scenarios such as emotion regulation and cognitive intervention.

To address these issues, this paper proposes an EEG generation model that integrates a Transformer architecture, a conditional VAE mechanism, and an adversarial learning strategy. The model introduces multi-dimensional loss constraints to achieve fidelity-oriented modeling of generated signals from temporal, spectral, and statistical perspectives, while incorporating emotion labels as conditional inputs to enable controllable generation of EEG segments with specific emotional attributes. The Transformer module effectively captures long-range temporal dependencies in EEG signals; the variational inference and reparameterization mechanisms enhance the interpretability of the latent space and the diversity of generated samples; and the discriminator, through adversarial feedback, improves the realism and structural consistency of synthetic data.

The main contributions of this work are as follows:

(1) Incorporation of emotion label conditioning to enhance semantic controllability of generated signals

Most existing EEG generation models are unconditional, making it difficult to generate target samples based on specified labels. This study incorporates conditional label embedding (one-hot) encoded emotion labels, concatenated in both the encoder and decoder stages, to achieve conditional modeling. This significantly improves the model’s ability to control emotional attributes, enabling the generation of EEG segments with target emotion characteristics while preserving signal authenticity.

(2) Integration of a Transformer encoder to improve temporal dependency modeling

Compared with traditional Convolutional Neural Networks (CNN) or Recurrent Neural Networks (RNN)’ architectures, Transformers are better suited for capturing long-range temporal dependencies and rhythmic variations in EEG signals. In this work, multi-layer Transformer encoders are integrated into the generator, leveraging self-attention mechanisms to model both local and global temporal features, thereby enhancing the representation of rhythmic structures in generated signals.

(3) Joint multi-dimensional structure-aware loss to improve multi-modal fidelity of generated samples

Given the complex structural characteristics of EEG signals in the temporal, spectral, and distributional domains, this study designs a multi-loss fusion strategy incorporating mean squared error (MSE), Pearson correlation, signal smoothness, power spectral mean squared error, and Kullback–Leibler (KL) divergence. This approach constrains generated samples from multiple perspectives, improving their structural consistency in morphology, spectrum, and statistical distribution.

## 2. Related Work

In recent years, generative models have attracted considerable attention in the modeling and synthesis of EEG signals. To address challenges such as the difficulty of EEG data acquisition, limited sample sizes, and distributional imbalance, researchers have increasingly explored the use of VAEs, GANs, and their hybrid architectures, aiming to improve the quality and representational capacity of synthesized signals. This section reviews related studies from two perspectives: VAE-based generative methods and GAN-based generative methods.

### 2.1. VAE-Based Methods for EEG Signal Generation

The VAE is a representative probabilistic generative model that achieves controllable sample generation by modeling the latent distribution of data. A typical VAE consists of an encoder, a decoder, and a latent space. The encoder maps the input signal to the distribution parameters of latent variables, from which a latent vector is sampled via the reparameterization trick, and the decoder reconstructs the original signal from this latent vector. The training objective comprises both a reconstruction error term and a KL divergence regularization term, thereby ensuring fidelity in signal reconstruction while optimizing the continuity and structural expressiveness of the latent space.

In the context of EEG signal generation, VAEs have been introduced primarily due to their ability to model the distributions of high-dimensional neural signals. In 2019, Aznan et al. [17] first proposed an end-to-end training approach to directly generate meaningful EEG data in the signal space, thereby improving the overall efficiency of EEG synthesis. In 2024, Cisotto et al. [18] proposed hvEEGNet, a hierarchical VAE trained with a DTW-based loss for multi-channel EEG reconstruction on BCI IV-2a (motor imagery), achieving subject-wise high-fidelity reconstructions. In 2022, Bethge et al. [19] designed the EEG2cVAE model for emotion recognition, enabling preliminary generation of EEG signals under different emotional states. In 2023, Li et al. [20] proposed the Causal Recurrent Variational Autoencoder (CR-VAE), which learns Granger causality graphs from multivariate time series and incorporates latent causal mechanisms into the data generation process. This approach not only generates EEG signals but also enhances transparency in the EEG generation process. In the same year, Tian et al. [21] introduced the Dual-Encoder Variational Autoencoder–Generative Adversarial Network (DEVAE-GAN), which integrates spatiotemporal features to address the issue of data sparsity in building robust EEG-based classification models.

Although VAEs have demonstrated certain advantages in EEG signal generation, the quality of the generated data often falls short compared to GAN-based models. Specifically, EEG segments generated by VAEs tend to contain noise interference and partially inaccurate waveform characteristics, which can adversely affect downstream applications. Furthermore, VAEs exhibit limited capability in capturing the complex spatiotemporal coupling features of EEG data. More critically, despite their promising generative performance in various EEG modeling tasks, a systematic understanding of how VAEs identify and reproduce specific EEG patterns remains lacking, which constrains their adoption, trustworthiness, and interpretability in both clinical and engineering applications.

### 2.2. GAN-Based Methods for EEG Signal Generation

GANs, first proposed by Goodfellow et al. [22] in 2014, are among the most representative generative models in recent years. Their core concept lies in training a generator and a discriminator in an adversarial framework, whereby the generator progressively produces data samples that more closely approximate the real distribution through a minimax game. GAN architecture has achieved groundbreaking advances in fields such as image synthesis and speech generation and have likewise attracted considerable interest from EEG researchers.

In 2018, Abdelfattah et al. [23] proposed a recurrent generative adversarial network (DCGAN), which follows the same design principles as conventional GANs but replaces the convolutional generator with a recurrent neural network, thereby enhancing the model’s ability to capture long-term dependencies in EEG data. In 2021, Fahimi et al. [24] introduced a DCGAN-based approach that employed a trained deep convolutional neural network to extract feature vectors from a subset of a target participant’s EEG data. These features were then combined with the original dataset to form an augmented training set, which improved the performance of EEG classifiers. In 2023, Dong et al. [12] proposed a DCGAN-based data augmentation method that introduced Gaussian noise into the generator and used convolutional neural networks to process EEG time series data. The authors analyzed the generated data using fast Fourier transform and continuous wavelet transform, finding strong similarity to real EEG signals. However, DCGAN-based architecture still suffers from typical issues such as training instability, gradient vanishing, and mode collapse—problems that become more pronounced in EEG applications due to high noise levels and substantial inter-subject variability.

To improve training stability and data quality, Arjovsky et al. [25] introduced the WGAN into EEG generation in 2017. By replacing the original cross-entropy loss with the Wasserstein distance, this model theoretically alleviates the problem of gradient discontinuity. In 2020, Panwar et al. [26] proposed WGAN-GP, incorporating a gradient penalty strategy to further enhance training stability. In 2020, Smith et al. [27] proposes a conditional GAN for 1D time-series synthesis (TSGAN), evaluated on 70+ univariate datasets from the UCR archive, and reports improved FID and TSTR/TRTS scores over WGAN baselines, indicating better realism and downstream utility under a conditional setting.

Although WGAN and its variants have, to some extent, addressed stability issues, several limitations remain:

(1) Lack of temporal modeling capability—Most EEG GAN models rely on one-dimensional convolutional architectures, which are inadequate for capturing long-range dependencies or rhythmically structured temporal patterns, impairing the accurate reconstruction of key frequency bands;
(2) Absence of label-conditioned control mechanisms—The original GAN framework is inherently unconditional, making it difficult to generate EEG samples with semantic attributes corresponding to specific emotional states or cognitive tasks;
(3) Neglect of structural loss constraints—GAN models typically optimize solely based on discriminator feedback and often omit explicit modeling of structural consistency in terms of temporal smoothness or spectral fidelity, resulting in waveform distortion or spectral drift in generated signals;
(4) Susceptibility to mode collapse—In multi-class emotion generation tasks, GANs often struggle to adequately cover the full data distribution across multiple emotional labels, leading to insufficient sample diversity.

Therefore, while GAN-based models exhibit promising potential for EEG signal generation, they still face significant challenges in maintaining frequency-domain consistency, class controllability, and temporal fidelity. To overcome these limitations, researchers have increasingly explored combining GANs with architectures such as VAEs and Transformers, leveraging both latent distribution modeling capabilities and adversarial learning mechanisms to more comprehensively capture the complex structure of EEG signals.

## 3. Materials and Methods

### 3.1. Dataset

This study employs the publicly available SEED (SJTU Emotion EEG Dataset) [28,29] as the primary data source for model training and evaluation. The SEED dataset was developed by Shanghai Jiao Tong University, with the aim of collecting multi-channel EEG signals through a film-elicitation paradigm to capture neural activity patterns associated with different emotional states. It has been widely used in research on emotion classification, affective computing, and EEG data modeling. Beyond SEED, this research additionally evaluates the proposed method on two datasets, SEED-FRA [30,31] and SEED-GER [30,31], providing culturally distinct cohorts for external validation.

The dataset comprises EEG recordings from 15 healthy participants with 7 males and 8 females aged 23.27 ± 2.37 years. In addition, this research evaluates on SEED-FRA with 5 males and 3 females aged 22.5 ± 2.78 years and SEED-GER with 7 males and 1 female aged 22.25 ± 1.98 years to protect personal privacy. Each participant took part in three experimental sessions, with at least a one-week interval between sessions to avoid emotional habituation effects. In each session, participants sequentially watched 15 emotion-eliciting video clips, each lasting approximately four minutes. The video content was pre-screened to induce three typical emotional states—positive, neutral, and negative—with five clips for each category. After viewing each clip, participants completed a self-assessment questionnaire to verify the consistency and validity of the elicited emotions.

EEG data were collected using the NeuroScan SynAmps2 system (Compumedics, Charlotte, NC, USA) with electrode placement following the international 10–20 system, covering 62 channels at an original sampling rate of 1000 Hz.

For each participant, the data from one experimental session are stored in a single file containing EEG recordings from 15 emotional trials. Each trial is stored as a matrix of dimensions [channels × time points], where N depends on the trial duration and sampling rate. Each trial is annotated with a clear emotion label, and label distributions are consistent across participants, ensuring standardization and comparability of the annotations.

### 3.2. Preprocessing

Prior to model training, the raw EEG signals were subjected to standard preprocessing steps to improve data consistency and facilitate model convergence. First, each EEG trial was reformatted to ensure a uniform dimension of [time × channels]. Subsequently, Z-score normalization was applied to each channel within a trial to eliminate amplitude discrepancies across channels.

EEG signals were down-sampled to 200 Hz, band-limited using a 0–45 Hz filter and cleaned via independent component analysis (ICA) to remove artifacts. To expand the dataset and capture temporal features, a sliding window segmentation was applied to the EEG signals, with a window length of 400 samples and a step size of 200 samples, a setting chosen to facilitate downstream classification tasks. Each segment inherited the emotion label of its parent trial, and labels were mapped to standardized category indices for use in conditional generative modeling.

### 3.3. Model

#### 3.3.1. Overall Architecture

**Figure 1:**
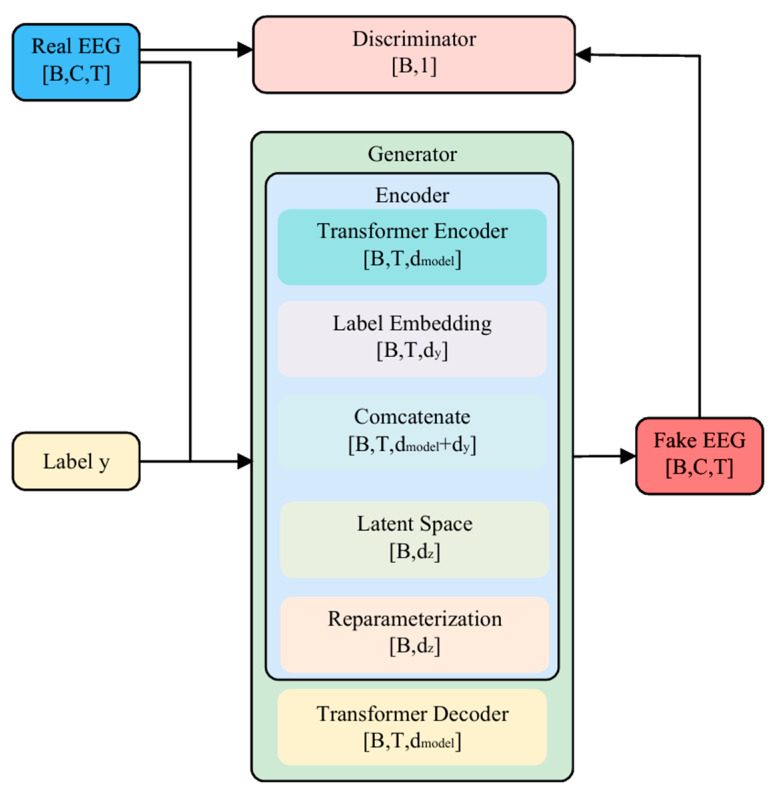
Trans-cVAE-GAN overall architecture.

To achieve high-quality EEG signal generation under explicit emotion label control, this research developed a deep generative model named Trans-cVAE-GAN, which integrates the concepts of cVAE, Transformer, and GAN. The model takes SEED EEG data as input, incorporates emotion labels for conditional modeling, and outputs synthetic EEG segments with the specified emotional attributes. The overall framework, illustrated in Figure 1, consists of four primary functional modules:

Transformer Encoder—Models the temporal dependencies in input EEG segments and extracts high-dimensional latent feature representations.

Latent Space Modeling and Reparameterization Module—Concatenates the encoder output with the embedded emotion label vector to obtain the mean and variance vectors of the latent variables, and samples a latent code via the reparameterization trick.

Conditional Decoder—Concatenates the sampled latent code with the emotion label, then passes it through a multilayer perceptron and linear mapping layers to produce the synthetic EEG signal.

Discriminator—Distinguishes between generated and real EEG signals, providing adversarial feedback to improve the realism and diversity of synthetic samples.

The model is trained end-to-end. The generator—comprising the encoder and decoder—optimizes for both reconstruction error and KL divergence in the latent space, while the discriminator engages in adversarial training with the generator to produce synthetic EEG signals that closely resemble real data while maintaining semantic consistency.

To further enhance the fidelity of generated samples in terms of temporal structure and spectral characteristics, the loss function incorporates additional multi-dimensional structural constraints, including Pearson correlation, temporal smoothness, and Welch power spectral distance. These are jointly optimized within the generator training process.

#### 3.3.2. Transformer Encoder

**Figure 2:**
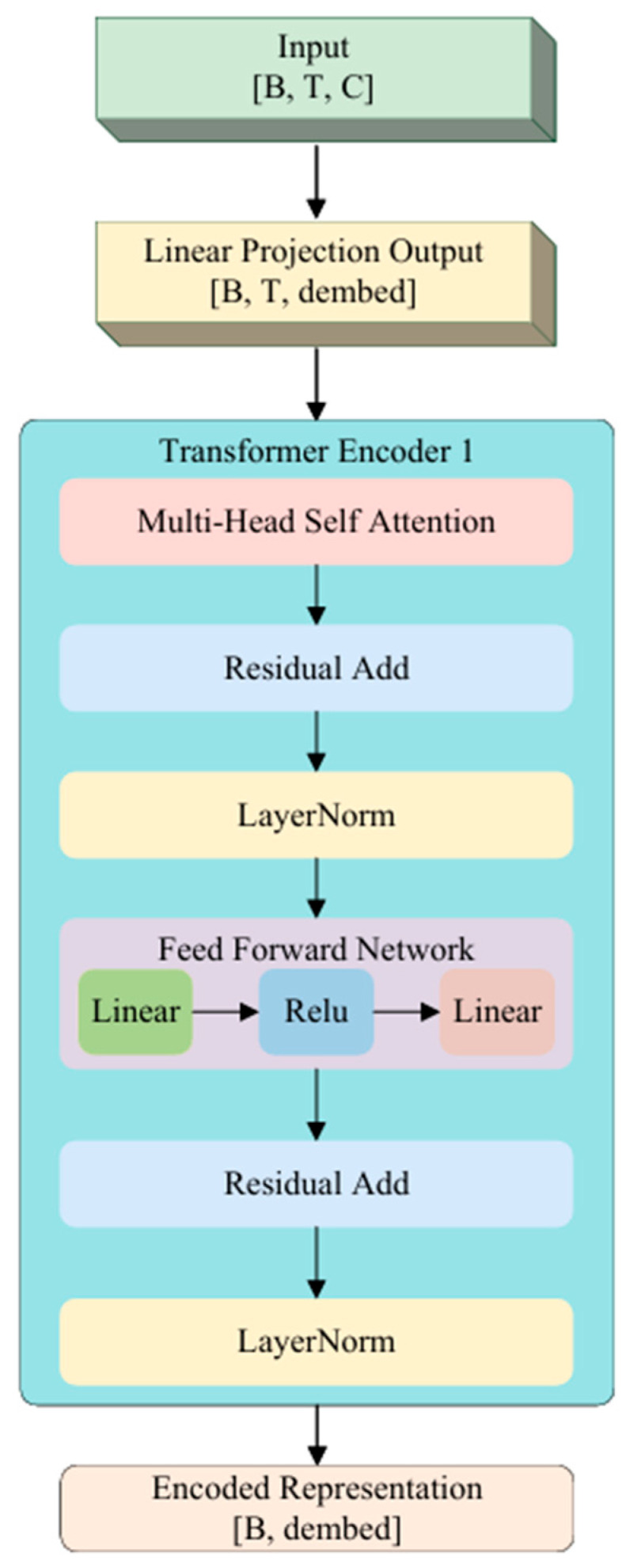
Transformer-based encoder architecture.

EEG signals are a highly nonstationary time series with strong temporal dependencies. To more effectively model their underlying temporal dynamics, this paper introduces a Transformer-based encoder, illustrated in Figure 2, into the generator’s encoder module. Compared with CNN or RNN, the Transformer can capture important dependencies across the entire temporal span via a multi-head attention mechanism, thereby enhancing the representational power and generalization ability of emotion-related EEG features.

In implementation, the input EEG data are shaped as [B,T,C], where B denotes batch size, T is the number of steps, and C is the number of channels. The input is first projected into an embedding space via a linear mapping layer, producing a time series representation of size $\left[B,T,d_{embed}\right]$. This sequence is then fed into a stack of two Transformer encoder layers, each consisting of multi-head self-attention and feed-forward networks, with residual connections and layer normalization applied for stable training.

The core advantage of the encoder lies in its ability to directly model interactions between any two time points, thereby capturing both local and global dynamic features of EEG activity. To obtain a fixed-length feature representation, the temporal dimension of the Transformer’s output is compressed via average pooling, resulting in a vector of size ${[B,d}_{embed}]$, denoted as $z_{input}$, which serves as the latent representation of the input segment.

This design enables the model to effectively encode the temporal patterns of EEG segments under different emotional states, providing a high-quality representation for subsequent latent space modeling and conditional generation.

#### 3.3.3. Latent Space Modeling and Label Embedding

In conditional generation tasks, latent space modeling must not only capture abstract representations of the input data but also explicitly incorporate external label information for class control. To this end, this paper introduces a label embedding mechanism into the encoder output and employs a variational inference strategy to model the latent variables, constructing a label-aware latent space representation.

Specifically, after passing through the Transformer encoder, an EEG segment is represented by a fixed-length vector $z_{input}\in R^{d}$. In parallel, the emotion label y∈{0,1,2} is converted into a conditional label embedding vector and mapped via a fully connected embedding layer into a representation $e_{y}$ of the same dimension as the encoder output. The two vectors, $z_{input}$ and $e_{y}$, are concatenated to form a joint representation ${[z}_{input},e_{y}]$, which serves as the input to latent space modeling.

This joint representation is fed into two independent fully connected networks to estimate the parameters of a Gaussian distribution over the latent variables: the mean vector $\mu \in R^{k}$ and the log-variance vector $log\sigma^{2}\in R^{k}$. To enable differentiable sampling, this research adopts the reparameterization trick, representing the sampling process as:

(1) $$z=\mu +\sigma \;⨀\;\;\epsilon ,\;\;\epsilon \in N\left(0,I\right)$$

where ⨀ denotes the Hadamard product and $\sigma =exp(0.5\cdot log\sigma^{2})$. This method avoids non-differentiability during backpropagation, allowing the latent variable z to participate in end-to-end neural network training.

Through this design, the model can learn the intrinsic data distribution while incorporating external emotion label information, thus enabling conditional latent space modeling that provides a structured prior for label-controlled decoding and generation.

### 3.4. Transformer Conditional Decoder

**Figure 3:**
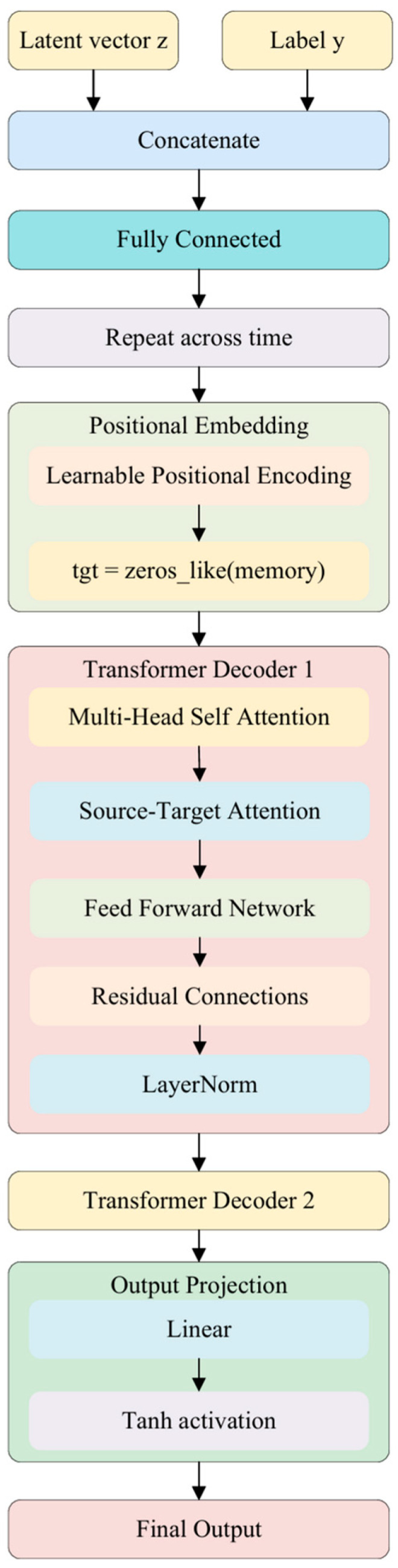
Transformer-based decoder architecture.

To generate EEG signals with explicit emotion label control, this research designs a Transformer-based conditional decoder, illustrated in Figure 3, that maps latent variables z from the latent space into multi-channel time series signals consistent with the morphology of real EEG. This module retains the decoding characteristics of variational generative models while incorporating the global modeling capacity of a Transformer decoder, thereby improving structural consistency and temporal fidelity in the synthesized signals.

In implementation, the latent variable $z\in R^{k}$ is concatenated with the conditional label embedding emotion label vector $y_{onehot}\in R^{n_{class}}$ to form a conditional vector $[z,y_{onehot}]\in R^{k+n_{class}}$. This vector is mapped via a fully connected layer to a target dimension and then replicated T times to construct a pseudo time sequence $M\in R^{T\times d_{embed}}$, which serves as the memory input to the Transformer decoder.

Simultaneously, a learnable positional encoding matrix $P\in R^{T\times d_{embed}}$ is introduced as the initial query (tgt) for the target sequence. During training, tgt is initialized as a zero tensor plus positional encodings, simulating the process of “decoding” a complete EEG sequence step-by-step from the latent variable.

The Transformer decoder consists of two stacked decoding layers, each comprising multi-head self-attention, source–target attention, and a feed-forward network, with residual connections and layer normalization. At each time step, the decoder combines the memory with its own historical outputs to iteratively refine the sequence representation. The final Transformer output is projected via a linear layer to the EEG channel dimension C, yielding an output of shape [B,T,C], consistent with the input format.

This conditional decoder offers three key advantages:

Direct embedding of label conditions within the decoding process;

Enhanced global modeling capacity through the Transformer architecture;

High retention of EEG temporal structure in the outputs, enabling high-fidelity, semantically controllable generation of emotion-related EEG data.

### 3.5. Discriminator Network

**Figure 4:**
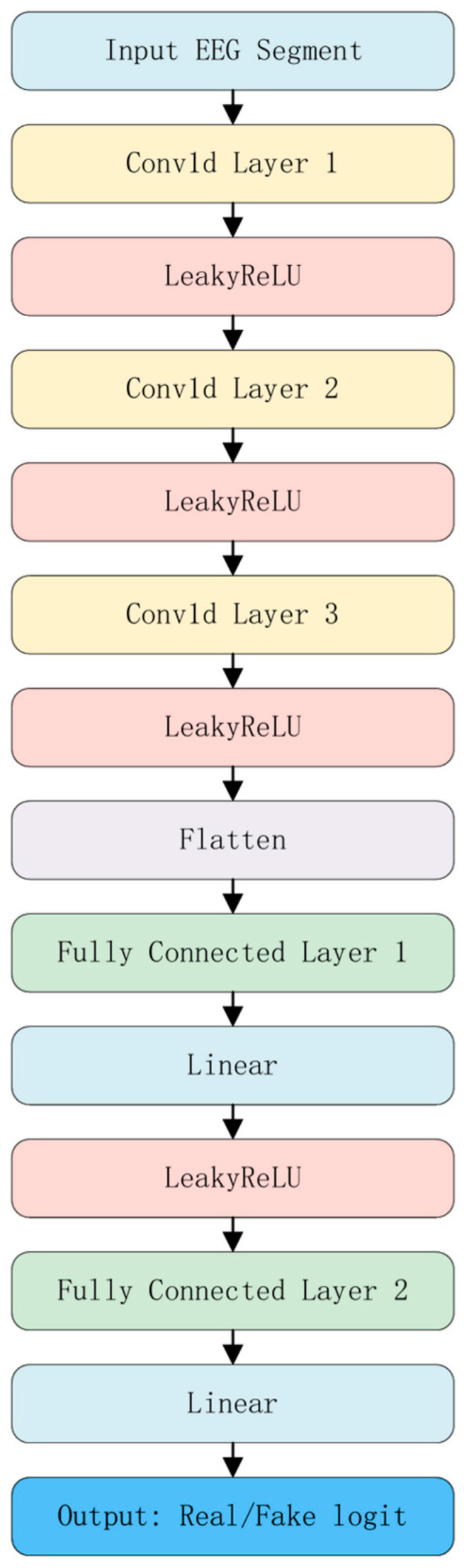
Discriminator network architecture.

To enhance the realism and distributional consistency of generated EEG signals, an adversarial learning mechanism is integrated into the generative framework. A lightweight one-dimensional convolutional discriminator network, illustrated in Figure 4, is designed to distinguish between generated and real EEG segments, thereby guiding the generator to produce high-quality EEG data with stronger neurophysiological characteristics.

The discriminator takes as input either generated or real EEG segments of shape [B,T,C], where T denotes the number of time steps and C denotes the number of channels. Before entering the discriminator, the input is transposed to [B,C,T] to accommodate the temporal sequence modeling requirements of one-dimensional convolution operations.

The network consists of three sequential one-dimensional convolutional layers with kernel sizes of 5, 5, and 3, respectively, and a stride of 2. Zero-padding is applied to maintain the temporal dimension. Each convolutional layer is followed by a LeakyReLU activation function to mitigate vanishing gradients and enhance expressiveness of features. The resulting high-dimensional temporal features are flattened into a vector via a Flatten operation and passed through two fully connected layers, ultimately outputting a scalar value that predicts the authenticity of the EEG segment.

The discriminator is trained to maximize the probability of correctly identifying real EEG signals while minimizing its error rate on generated EEG signals. Through this adversarial process, the generator is driven to produce synthetic samples that are increasingly challenging for the discriminator to classify. During training, the generator and discriminator are updated alternately, forming a game-theoretic loop that stably improves both the quality of generated data and the discriminator’s capacity.

Overall, the proposed discriminator is compact and efficient, capable of capturing subtle differences between generated and real EEG in both local temporal patterns and global statistical distributions. This provides the generator with effective training feedback, enhancing the expressive power and practical value of the overall generative system.

### 3.6. Loss Function and Joint Optimization Strategy

To improve the structural, semantic, and spectral fidelity of generated EEG signals, this research designs a multi-objective joint optimization strategy that integrates the reconstruction objectives of the VAE, the authenticity discrimination objective of the GAN, and multiple structural consistency metrics into a unified training framework. The overall loss function comprises the following six components:

(1) Reconstruction Loss

Measures the amplitude difference between generated and real EEG segments in the time domain using Mean Squared Error (MSE):

(2) $$L_{recon}=\frac{1}{N}\sum_{i=1}^{N}{\left\|\;{\hat{x}}^{\left(i\right)}-x^{\left(i\right)}\right\|}_{2}^{2}$$

(2) KL Divergence Loss

Regularizes the distribution of latent variables to approximate a standard normal distribution, ensuring continuity and sample ability of the latent space:

(3) $$L_{KL}=-\frac{1}{2}\sum_{j=1}^{k}\left(1+log\sigma_{j}^{2}-\mu_{j}^{2}-\sigma_{j}^{2}\right)$$

(3) Adversarial Loss

Measures the generator’s ability to fool the discriminator, guiding it toward the real data distribution. This research adopts the standard binary cross-entropy form:

(4) $$L_{adv}=E_{\hat{x}}[-logD(\hat{x})]$$

(4) Pearson Correlation Loss

Evaluates morphological trend similarity between generated and real signals by computing the average channel-wise correlation per sample and taking its complement:

(5) $$L_{corr}=1-corr(\hat{x},x)$$

(5) Smoothness Loss

Constrains temporal smoothness of generated signals by penalizing large variations between adjacent time steps:

(6) $$L_{smooth}=\frac{1}{T-1}\sum_{t=1}^{T-1}{\left\|\;{\hat{x}}_{t}-{\hat{x}}_{t+1}\right\|}_{2}^{2}$$

(6) Power Spectrum Consistency Loss

Enhances spectral-domain structural consistency by comparing the power spectral density (PSD) of generated and real signals. The Welch method is used to estimate PSD, and the MSE between the two spectra is computed.

To prevent imbalance among the multiple objectives from negatively affecting training, a dynamic weighting mechanism is introduced. Each loss component is normalized, and its weight is automatically allocated via a Softmax distribution with bounded values, ensuring that all objectives receive sufficient optimization during training. The total generator loss is defined as:

To balance the six objectives during training, this research adopts a batch-wise dynamic weighting scheme. At each batch, the scalar losses $L_{i}$ (reconstruction, KL, adversarial, Pearson, smoothness, spectrum) are z-scored within the batch, passed through a softmax, then clipped to [0.1, 0.4] and renormalized to sum to one. This research computed the weights under no-grad. The weights are not learnable and require no initialization, which keeps optimization numerically stable and prevents any single objective from dominating. With six terms, the post-clipping bounds imply a maximum per-term contribution of 0.4/(0.4 + 5 × 0.1) = 0.444 and a minimum of 0.1/(0.4 + 5 × 0.1) = 0.111. The generator loss is

(7) $$L_{gen}=\sum_{i=1}^{6}\alpha_{i}\cdot L_{i}$$

where $\alpha_{i}$ are the dynamically computed weights.

The generator and discriminator are trained alternately. In each iteration, the discriminator is updated first to improve its ability to distinguish between real and generated data, followed by two updates to the generator to enhance both reconstruction accuracy and its capacity to fool the discriminator.

Through this multi-objective joint loss design and adversarial optimization strategy, the model can accurately reconstruct EEG segments in the temporal amplitude domain while closely matching real signals in morphology, spectrum, and statistical structure—achieving multi-perspective, high-consistency generation of emotion-related EEG signals.

## 4. Experimental Design and Results Analysis

### 4.1. Experimental Environment

All experiments were conducted on a Windows server equipped with an NVIDIA RTX 4060Ti GPU (NVIDIA Corporation, Santa Clara, CA, USA). The operating system was Windows 11 (Microsoft Corporation, Redmond, WA, USA), the primary development environment was Python 3.10, and the deep learning framework used was PyTorch 2.0.1. Additional computational and signal processing libraries included NumPy 1.24.4, SciPy 1.10.1, and scikit-learn 1.3.2. To ensure experimental stability, all random processes were initialized with fixed random seeds.

### 4.2. Implementation Details and Hyperparameters

The training configuration as below: a Transformer encoder with 2 layers, 4 heads, 256-dim embedding, a VAE latent dimension of 128, dynamic loss weighting (softmax on z-scored losses clamped to [0.1, 0.4]), Adam optimizers (generator lr = 1 × 10^−3^, discriminator lr = 1 × 10^−4^, $\beta_{1}=0.9$, $\beta_{1}=0.999$, no learning-rate schedule, batch size 64, and 100 epochs.

### 4.3. Evaluation Metrics

To comprehensively evaluate the performance of the proposed EEG signal generation model in terms of realism, structural consistency, and spectral preservation, this research designed multiple objective evaluation metrics from the perspectives of statistical correlation, frequency-domain consistency, information divergence, and feature-space distribution.

#### 4.3.1. Pearson Correlation Coefficient

The Pearson correlation coefficient is used to measure the channel-level linear correlation between generated signals and real signals, defined as:

(8) $$r_{xy}=\frac{\sum_{i=1}^{n}\left(x_{i}-\bar{x}\right){\left(y_{i}-\bar{y}\right)}^{2}}{\sqrt{\sum_{i=1}^{n}{\left(x_{i}-\bar{x}\right)}^{2}}\cdot \sqrt{\sum_{i=1}^{n}{\left(y_{i}-\bar{y}\right)}^{2}}}$$

where $x_{i}$ and $y_{i}$ represent the i-th sampling points of the real and generated signals, and $\bar{x}$ and $\bar{y}$ denote their respective meaning. This metric reflects whether the generated signals preserve the temporal morphology of the real signals.

#### 4.3.2. Spearman’s Rank Correlation Coefficient

To further assess the nonlinear relationship between generated and real signals, Spearman’s rank correlation coefficient is introduced. It is calculated based on the rank differences in the signal values and is suitable for evaluating non-normal or nonlinear signal relationships:

(9) $$\rho =1-\frac{6\sum d_{i}^{2}}{n(n^{2}-1)}$$

where $d_{i}$ is the rank difference in the i-th sampling point in the two signals, and n is the number of sampling points.

#### 4.3.3. Kullback–Leibler Divergence

KL divergence is used to quantify the informational difference between the spectral distributions of real and generated signals, defined as:

(10) $$D_{KL}(P\left\|Q\right)=\sum_{i=1}^{n}P(i)log(\frac{P(i)}{Q(i)})$$

where P(i) and Q(i) are the normalized power spectral values of the real and generated signals at the i-th frequency bin.

In subsequent experiments, this research reports average metric values across all channels and emotion categories and compares them against reference baselines to systematically evaluate the realism, spectral fidelity, and label controllability of the proposed model.

#### 4.3.4. Fréchet Distance

To assess multivariate distributional similarity beyond pointwise shape and spectral overlap, this research computes the Fréchet distance (FID) between Gaussian embeddings of windowed, channel-wise EEG features extracted from real and generated signals. Per channel, the waveform is segmented into short windows of 0.30 s with 0.15 s hop; for each window this research form a feature vector comprising five band-power integrals (0.5–4, 4–8, 8–13, 13–30, 30–45 Hz; log-energy), the spectral centroid and spectral entropy computed from a Welch PSD normalized over 0.5–45 Hz, the time-domain RMS, and AR(4) coefficients estimated via Yule–Walker. Real and generated feature sets are pooled and z-normalized feature-wise, then split to estimate means $\mu_{1}$, $\mu_{2}$ and covariances $C_{1}$, $C_{2}$; covariances are symmetrized and diagonally shrunk to ensure positive semi-definiteness, and the matrix square root uses an SVD of $C_{1}C_{2}$. The resulting Fréchet (Gaussian) distance is

(11) FIDX,Z=||μ1−μ2||2+Tr(C1+C2−2C1C212)

reported per channel (lower is better). By summarizing joint statistics of short-term time–frequency descriptors, FID complements Pearson/Spearman and KL/PSD-MSE, providing a distribution-level criterion for alignment between real and synthesized EEG.

#### 4.3.5. Mean Squared Error

To assess frequency-domain similarity in a phase-agnostic manner, this researcher compares the Welch PSDs of real and generated signals per channel. For each channel, PSDs are computed with a 2.0 s Hamming window, 50% overlap, and $N_{fft}=1024$, then band-limited to 0.5–45 Hz. To remove scale effects, each PSD is L^1^-normalized over the band to form a probability-like spectral envelope, ${\tilde{P}}_{x}\left(f\right)=P_{x}(f)/\int_{0.5}^{45}P_{x}(u)du$ and ${\tilde{P}}_{z}\left(f\right)=P_{z}(f)/\int_{0.5}^{45}P_{z}(u)du$. Denoting the Welch frequency grid within [0.5,45] Hz by ${\left\{f_{i}\right\}}_{i=1}^{M}$, the PSD-MSE is the discrete mean-squared error between the normalized envelopes:

(12) $$MSE\left(x,z\right)=\frac{1}{M}\sum_{i=1}^{M}{\left({\tilde{P}}_{x}\left(f_{i}\right)-{\tilde{P}}_{z}\left(f_{i}\right)\right)}^{2}\;$$

where lower values indicate closer spectral envelopes. PSD-MSE complements time-domain shape metrics and divergence measures by specifically quantifying frequency-wise amplitude redistribution while remaining insensitive to phase.

#### 4.3.6. Earth Mover’s Distance

To quantify frequency-domain discrepancy in a phase-agnostic way, this research computes the 1-D Earth Mover’s Distance (EMD) between the Welch PSDs of real and generated signals on each channel. For each channel, a zero-phase Welch estimate is obtained and restricted to 0.5–45 Hz. The resulting spectra $P_{x}\left(f\right)\;$ and $P_{z}\left(f\right)$ are L^1^-normalized to form probability densities over frequency, from which cumulative distribution functions $F_{x}\left(f\right)$ and $F_{z}\left(f\right)$ are constructed. The EMD is then the area between these CDFs over the band:

(13) $$EMD\left(x,z\right)=\int_{0.5}^{45}\left|F_{x}\left(f\right)-F_{z}\left(f\right)\right|df$$

reported per channel in units of Hz. Because it compares spectral envelopes rather than pointwise amplitudes, EMD complements Pearson/Spearman and PSD-MSE/KL by capturing frequency-wise mass shifts that reflect band-power redistribution between real and synthesized EEG.

#### 4.3.7. Classification Consistency

To assess semantic consistency from a downstream perspective, this research adopts a frozen-classifier congruence protocol. A CNN baseline is trained only on real EEG to learn mappings from signals to emotion labels; after training, the classifier is frozen and used to score generated segments conditioned on the same labels. If synthesized signals preserve label-relevant structure, performance on generated data should approach that on real data.

This research reports accuracy, precision, recall, F1-score, and AUC. To avoid cross-subject leakage, this research uses within-subject, trial-level splits on each dataset: trials pooled across the three sessions are stratified by label and partitioned 80%/10%/10% into train/validation/test; all windows inherit their parent trial’s split. The frozen classifier’s hyper-parameters and decision thresholds are fixed by the validation set; the test set remains untouched and real-only.

This protocol evaluates whether generated samples are interpretable by a classifier trained on real data (semantic congruence) without conflating effects from joint training. The augmentation study—where generated segments are added to the real training set while validation/test remain real-only will be reported in Section 4.3, isolating its impact on generalization.

### 4.4. Experimental Results and Analysis

#### 4.4.1. Time-Domain Comparison

**Figure 5:**
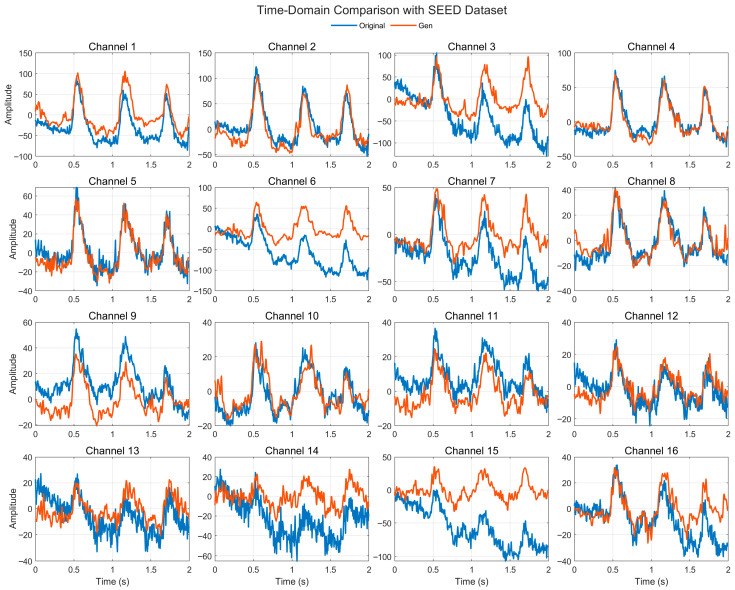
Time-domain signal comparison for channel 0–16 with SEED Dataset.

Figure 5 illustrates the time-domain comparison between real and generated EEG signals across 16 representative channels from the SEED dataset. Overall, the generated signals reproduce the major peaks, troughs, and envelope fluctuations of the real EEG, showing consistent temporal dynamics. In most channels, the phases of oscillatory components are well aligned, while in a few channels (e.g., 3, 6, and 15) the generated signals show amplitude compression or upward offset. Despite these local deviations, the generated signals preserve the overall waveform morphology and cross-channel coherence, indicating that the proposed model effectively captures both global temporal dependencies and local oscillatory structure, which are essential for emotion-related EEG generation.

**Figure 6:**
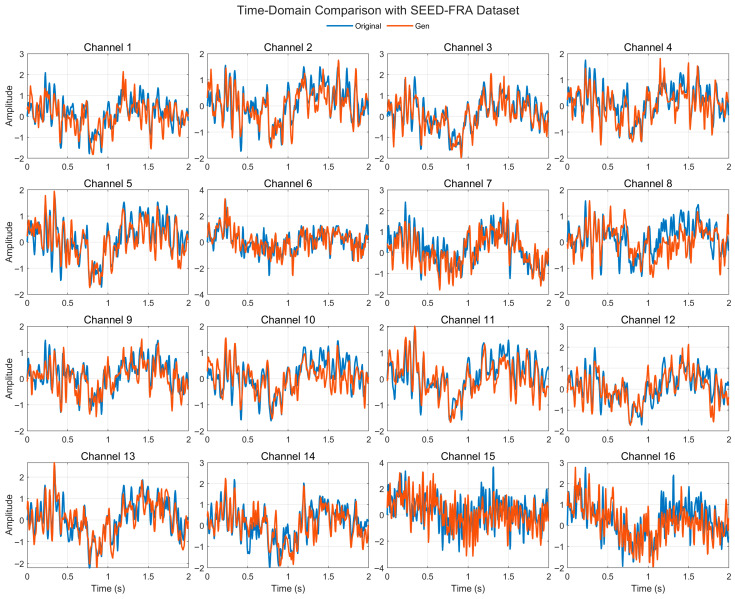
Time-domain signal comparison for channel 0–16 with SEED-FRA Dataset.

Figure 6 illustrates the time-domain comparison between real and generated EEG signals across 16 representative channels from the SEED-FRA dataset. The generated traces closely follow the real waveforms with well-aligned phases and comparable short-term fluctuations; most channels exhibit tight overlaps within the [−2, 3] a.u. range implied by z-scaling. A few channels show local amplitude compression or slight baseline offsets, yet the overall waveform morphology and cross-channel coherence are preserved. These observations indicate that the model generalizes SEED-FRA by capturing both global temporal dependencies and local oscillatory structure under a distinct subject/language cohort.

**Figure 7:**
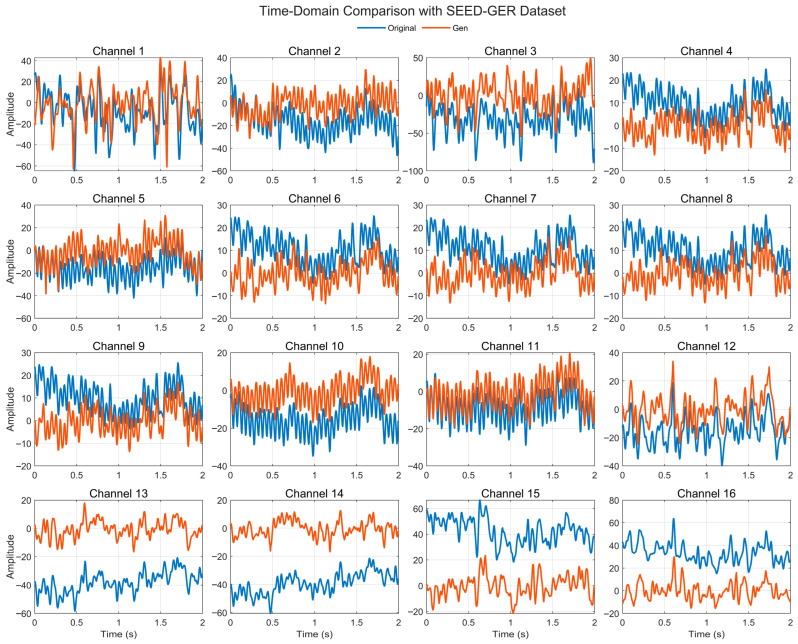
Time-domain signal comparison for channel 0–16 with SEED-GER Dataset.

Figure 7 illustrates the time-domain comparison between real and generated EEG signals across 16 representative channels from the SEED-GER dataset. The generated traces generally follow the rhythm and envelope of the real waveforms, with comparable short-term oscillations and visible peak–trough sequences. Several channels (e.g., 2, 3, 6, 13–16) exhibit baseline offsets and amplitude scaling relative to the real signals, whereas other channels show closer overlap. Despite these deviations, the generated signals preserve the overall waveform morphology and temporal patterning across channels, indicating that the model transfers to SEED-GER while retaining task-relevant dynamics. The residual offsets likely reflect dataset-specific amplitude/reference characteristics and could be further reduced with amplitude calibration or re-referencing.

#### 4.4.2. Pearson Correlation Analysis

**Figure 8:**
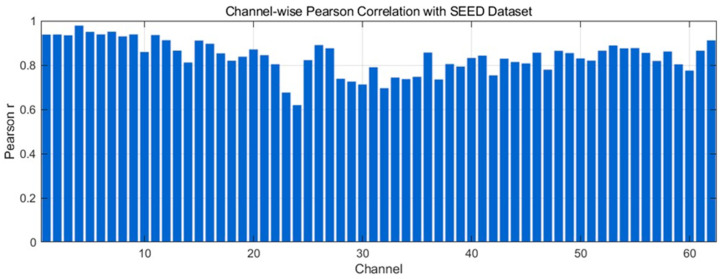
Channel-wise Pearson correlation distribution with SEED Dataset.

Figure 8 presents the channel-wise Pearson correlation coefficients between generated and real EEG signals from the SEED dataset. Most channels exhibit high correlations, with the majority ≥0.8 and many approaching 0.9, indicating strong preservation of temporal morphology. A small number of channels lie in the 0.7–0.8 range, suggesting mild amplitude/phase mismatches without altering the overall trends. Overall, the distribution shows that the proposed model robustly reproduces channel-specific temporal structures and maintains cross-channel consistency with high fidelity.

**Figure 9:**
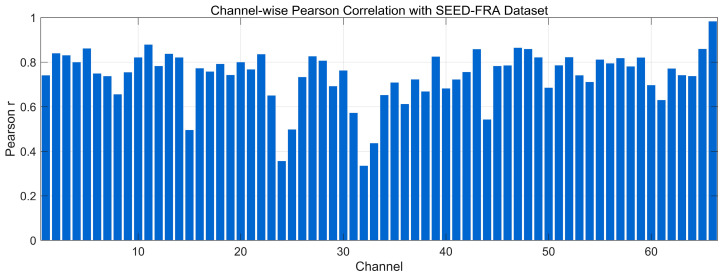
Channel-wise Pearson correlation distribution with SEED-FRA Dataset.

Figure 9 presents the channel-wise Pearson correlation between real and generated EEG on the SEED-FRA dataset. Most channels fall in the 0.70–0.85 range, with several channels approaching or exceeding 0.90, indicating good preservation of temporal morphology for the majority of channels. A subset of channels lies around 0.50–0.70, and a small cluster dips below 0.50 (mid-index channels), reflecting localized amplitude/phase mismatches that may stem from noise or corpus-specific variability. Overall, the distribution suggests that the model maintains channel-specific waveform structure on SEED-FRA while leaving room for improvement on a few channels with lower alignment.

**Figure 10:**
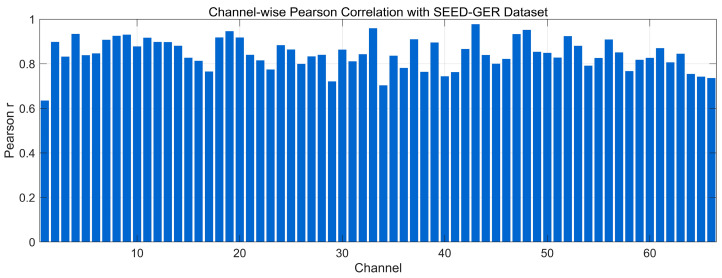
Channel-wise Pearson correlation distribution with SEED-GER Dataset.

Figure 10 presents the channel-wise Pearson correlation between real and generated EEG on the SEED-GER dataset. Most channels exhibit high correlations (≥0.80), with many approaching 0.90–0.95, indicating strong preservation of time-domain morphology across channels. A smaller subset lies around 0.70–0.80, reflecting mild amplitude/phase mismatches consistent with the time-domain offsets observed in several channels, yet without altering the overall trend structure. Overall, the distribution suggests that the model maintains channel-specific waveform similarity on SEED-GER with uniformly high alignment and only limited room for improvement on a few channels.

#### 4.4.3. Spearman Correlation Analysis

**Figure 11:**
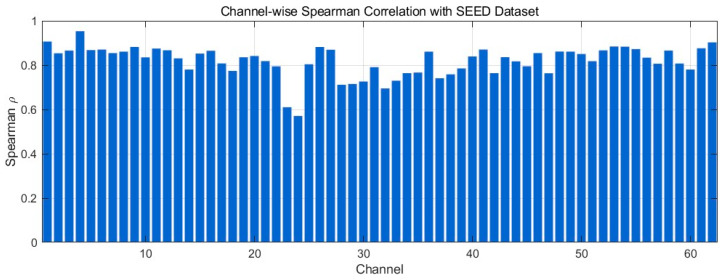
Channel-wise Spearman correlation comparison with SEED Dataset.

Figure 11 presents the channel-wise Spearman correlation between generated and real EEG signals from the SEED dataset. Most channels exhibit high coefficients, with the majority in the 0.80–0.90 range and several approaching 0.95, indicating preservation of monotonic rank-order relationships and local fluctuation patterns. A small subset lies around 0.70–0.80, suggesting minor amplitude/phase mismatches without altering overall trends; very low values (<0.50) are not observed. Taken together, these results indicate that the generated EEG maintains the nonlinear structural characteristics of real EEG, supporting robustness in temporal-morphology reconstruction.

**Figure 12:**
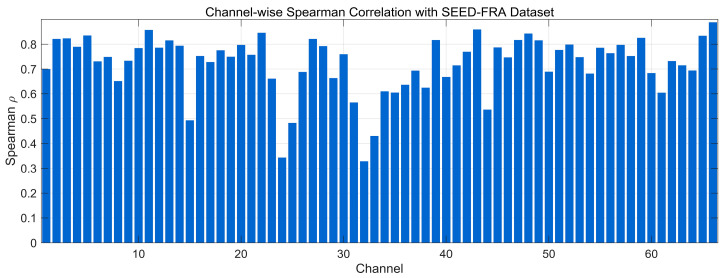
Channel-wise Spearman correlation comparison with SEED-FRA Dataset.

Figure 12 presents the channel-wise Spearman correlation between generated and real EEG on the SEED-FRA dataset. Most channels lie in the 0.70–0.85 range, indicating that the model preserves monotonic rank-order relationships and local fluctuation patterns. Several channels fall around 0.55–0.70, and a small cluster dips below 0.50 (mid-index channels), reflecting localized amplitude/phase mismatches likely driven by noise or corpus-specific variability. A few channels approach 0.85–0.90 toward the upper end, showing strong alignment. Overall, the distribution suggests good nonlinear structural consistency on SEED-FRA, with room to improve alignment for the subset of lower-scoring channels.

**Figure 13:**
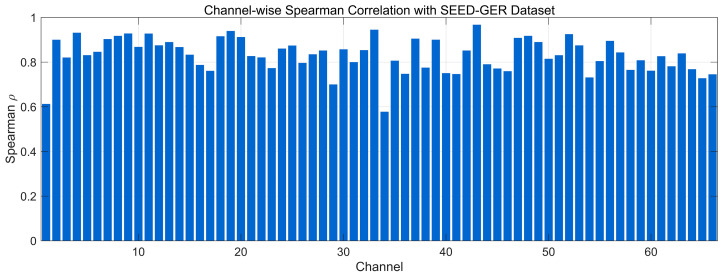
Channel-wise Spearman correlation comparison with SEED-GER Dataset.

Figure 13 presents the channel-wise Spearman correlation between generated and real EEG on the SEED-GER dataset. Most channels exhibit high coefficients (≈0.80–0.90), with several approaching 0.95, indicating strong preservation of monotonic rank-order relationships and local fluctuation patterns. A smaller subset lies around 0.70–0.80, consistent with mild amplitude/phase mismatches observed in a few channels, yet without altering overall trends. Overall, the distribution suggests uniformly good nonlinear structural alignment on SEED-GER, with limited room for improvement on lower-scoring channels.

#### 4.4.4. Spectral Distribution Information Divergence Analysis

**Figure 14:**
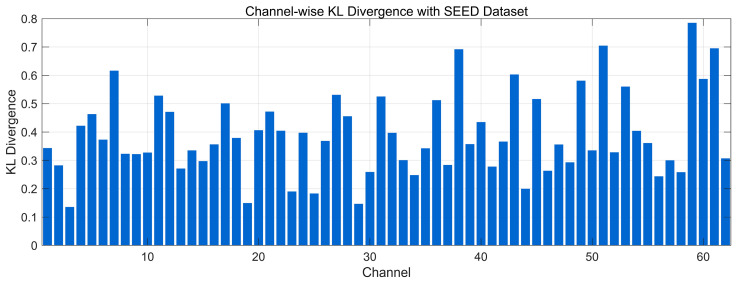
Channel-wise KL divergence comparison with SEED Dataset.

Figure 14 illustrates the channel-wise KL divergence between the spectral distributions (0.5–45 Hz) of generated and real EEG signals from the SEED dataset. Most channels lie around 0.25–0.50, indicating acceptable preservation of frequency-domain structure for the majority of channels. A subset shows higher divergence, with several channels reaching ~0.60–0.75 (and a few approaching 0.80), suggesting localized spectral-envelope mismatches (e.g., band-power shifts) that may arise from noise or subject-specific variability. Overall, the distribution indicates broadly similar spectral structure with pockets of discrepancy, without evidence of severe global distortions.

**Figure 15:**
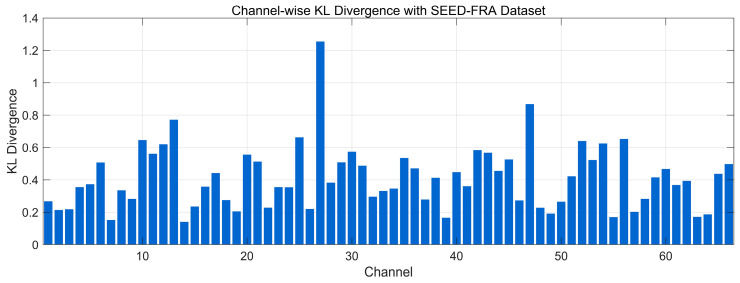
Channel-wise KL divergence comparison with SEED-FRA Dataset.

Figure 15 illustrates the channel-wise KL divergence between the 0.5–45 Hz spectral distributions of real and generated EEG on the SEED-FRA dataset. Most channels fall in the low-to-moderate range (~0.20–0.60), indicating acceptable preservation of frequency-domain structure for the majority of channels. Several channels show elevated divergence (~0.70–0.95) and isolated peaks exceed 1.0, suggesting localized spectral-envelope mismatches (e.g., band-power shifts or cohort-specific variability). Overall, the distribution points to broadly similar spectral profiles on SEED-FRA with a small subset of channels requiring further calibration to reduce divergence.

**Figure 16:**
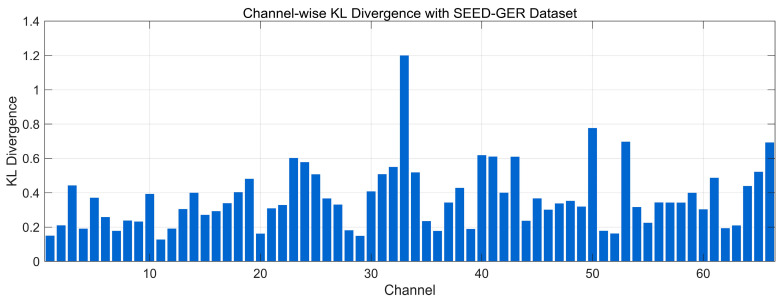
Channel-wise KL divergence comparison with SEED-GER Dataset.

Figure 16 illustrates the channel-wise KL divergence between the 0.5–45 Hz spectral distributions of real and generated EEG on the SEED-GER dataset. Most channels lie in a low–moderate range (~0.20–0.60), indicating broadly preserved spectral envelopes, while several channels rise to ~0.70–0.90 and isolated peaks approach ~1.2, reflecting localized band-power shifts or cohort-specific variability. Overall, the distribution suggests generally similar frequency-domain structure on SEED-GER with a small subset of channels exhibiting higher divergence that could benefit from additional calibration.

#### 4.4.5. Frequency-Domain Consistency Analysis

**Figure 17:**
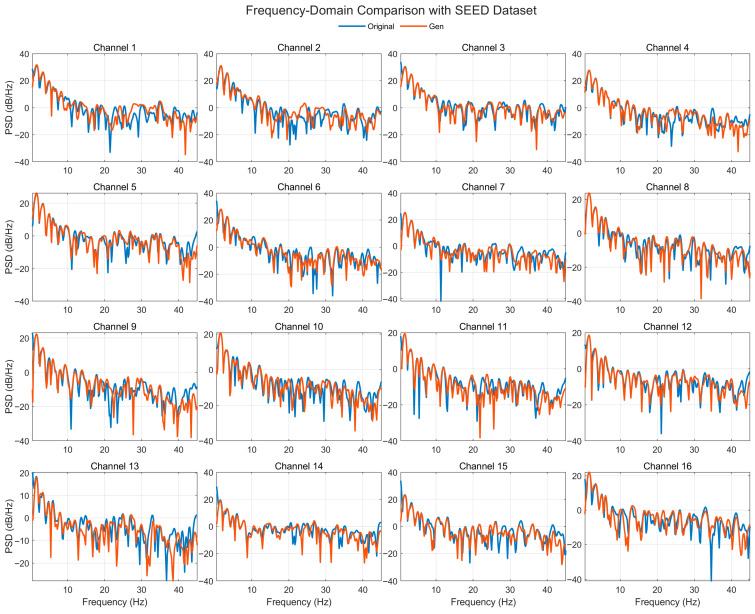
Frequency power spectrum comparison for channel 1–16 with SEED Dataset.

Figure 17 compares the Welch PSDs (0.5–45 Hz, dB/Hz) of real and generated EEG across 16 representative channels from the SEED dataset. The generated spectra closely follow the 1/f-like slope and band-limited peaks of the real signals, with clear agreement in the δ (0.5–4 Hz), θ (4–8 Hz), and α (8–13 Hz) bands. The β (13–30 Hz) and low-γ (30–45 Hz) ranges are broadly aligned, though several channels (e.g., 6, 10, and 15) show mild power offsets or slightly deeper/narrower notches around specific peaks. Despite these local differences, peak locations and the overall spectral envelope are preserved, and no spurious high-frequency energy is observed. These results indicate that the proposed model stably reconstructs frequency-domain structure, capturing global rhythmic trends together with channel-specific spectral details relevant for emotion-related analysis.

**Figure 18:**
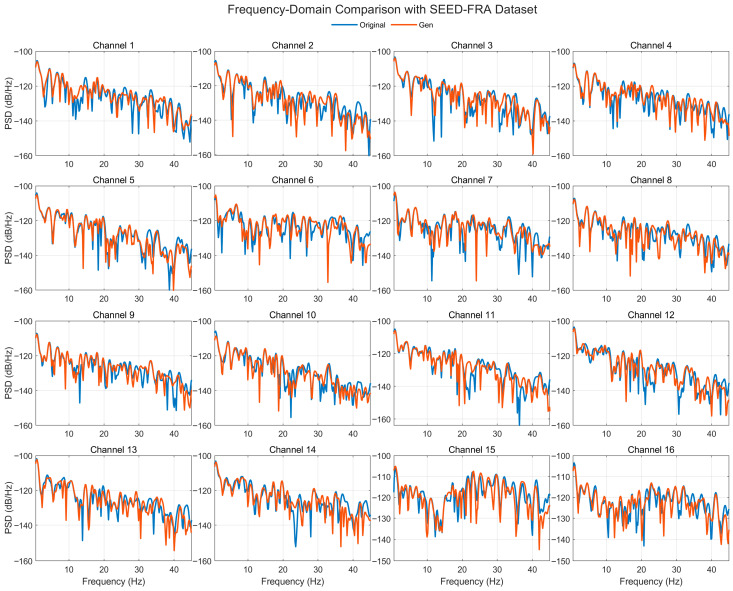
Frequency power spectrum comparison for channel 1–16 with SEED-FRA Dataset.

Figure 18 compares the Welch PSDs (0.5–45 Hz, dB/Hz) of real and generated EEG across 16 representative channels from the SEED-FRA dataset. The generated spectra closely track the real 1/f-like slope and band-limited peaks, with clear agreement in the δ (0.5–4 Hz), θ (4–8 Hz), and α (8–13 Hz) ranges. The β (13–30 Hz) and low-γ (30–45 Hz) bands are broadly aligned as well, though several channels show mild level offsets (a few dB) or slightly deeper/narrower notches at specific frequencies. Despite these local differences, peak locations and the overall spectral envelope are preserved, and no spurious high-frequency energy is observed. These results indicate that the model generalizes SEED-FRA while stably reconstructing frequency-domain structure relevant for emotion-related analysis.

**Figure 19:**
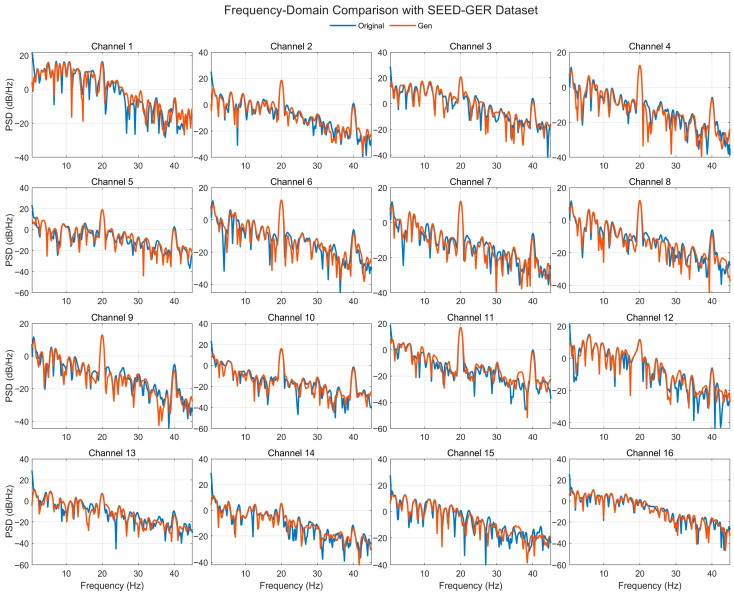
Frequency power spectrum comparison for channel 1–16 with SEED-GER Dataset.

Figure 19 compares the Welch PSDs (0.5–45 Hz, dB/Hz) of real and generated EEG across 16 representative channels from the SEED-GER dataset. The generated spectra generally track the real 1/f slope and band-limited peaks with clear agreement in δ (0.5–4 Hz), θ (4–8 Hz), and α (8–13 Hz), and broadly aligned energy in β (13–30 Hz) and low-γ (30–45 Hz). Several channels exhibit level offsets of a few dB and sharper or slightly shifted notches/peaks around mid-beta frequencies, while others show near-overlap across the band. Despite these local differences, peak locations and the overall spectral envelope are preserved, and no spurious high-frequency energy is observed, indicating that the model transfers to SEED-GER while stably reconstructing frequency-domain structure relevant for emotion-related analysis.

#### 4.4.6. Feature-Space Distribution Visualization Analysis

**Figure 20:**
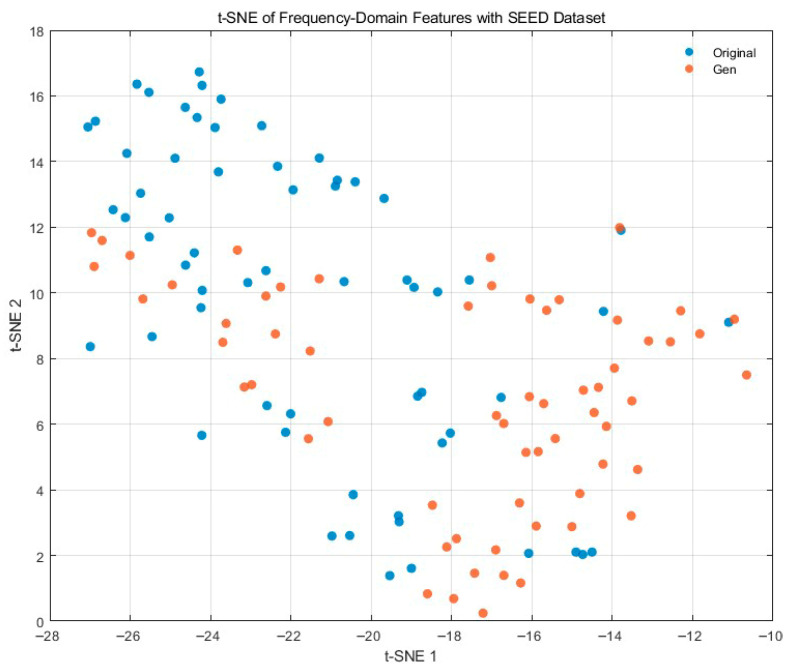
t-SNE of Frequency-Domain Features distribution with SEED Dataset.

Figure 20 presents a t-SNE visualization of PSD-based frequency-domain features (0.5–45 Hz) for real (blue) and generated (orange) EEG from the SEED dataset. The two sets occupy a similar manifold with comparable spread, and there is substantial local overlap, indicating preservation of global feature-space structure. At the same time, the generated points show a modest centroid shift (primarily along the t-SNE-2 axis) relative to the real ones, consistent with a small domain offset rather than mode collapse. Overall, the neighborhood structure is maintained, and no isolated or collapsed clusters are observed, supporting that the synthesized signals capture physiologically meaningful spectral statistics while exhibiting a mild distributional shift that could be further reduced by calibration or domain alignment.

**Figure 21:**
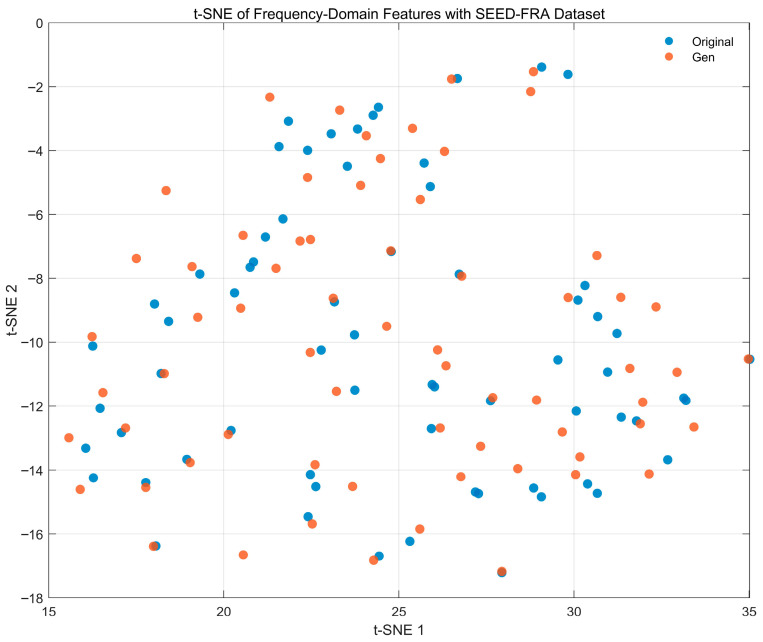
t-SNE of Frequency-Domain Features distribution with SEED-FRA Dataset.

Figure 21 presents a t-SNE visualization of PSD-based frequency-domain features (0.5–45 Hz) for real (blue) and generated (orange) EEG from the SEED-FRA dataset. The two sets occupy a similar manifold with comparable spread, showing substantial local overlap across the plane; several mixed neighborhoods appear, including the right-hand cluster, indicating that the generator preserves the global structure of the spectral feature space. A small, nearly uniform shift in some orange points relative to nearby blue points can be seen (without isolated or collapsed clusters), suggesting a mild domain offset rather than mode collapse. Overall, the neighborhood geometry is maintained, supporting that the synthesized signals capture physiologically meaningful spectral statistics on SEED-FRA.

**Figure 22:**
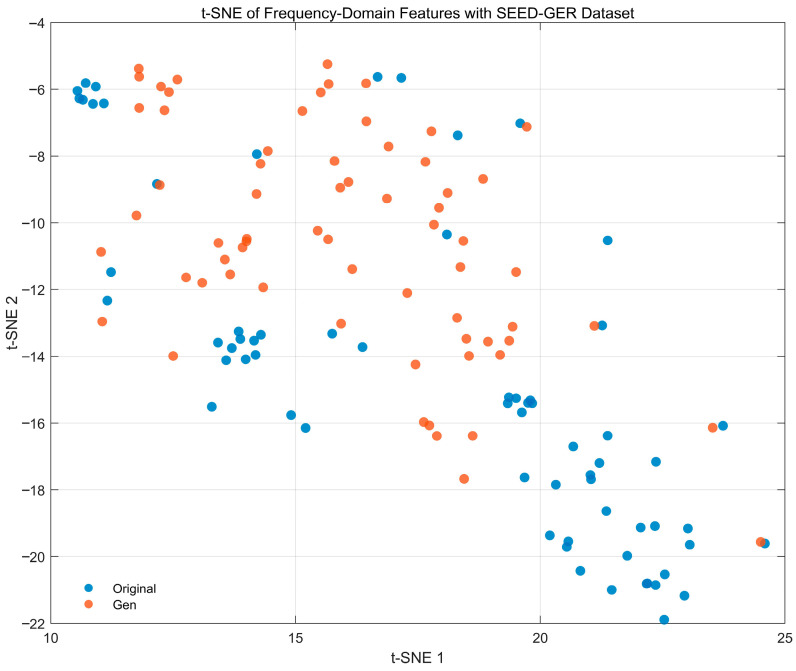
t-SNE of Frequency-Domain Features distribution with SEED-GER Dataset.

Figure 22 presents a t-SNE visualization of PSD-based frequency-domain features (0.5–45 Hz) for real (blue) and generated (orange) EEG from the SEED-GER dataset. The two sets trace a similar global manifold but exhibit a clear centroid shift along the diagonal (t-SNE-1/2) direction, yielding partially separated bands rather than mixed neighborhoods. This pattern indicates a systematic domain offset in spectral statistics—consistent with the level/offset differences observed in the GER spectra—while mode coverage is preserved (no collapsed or isolated clusters). Overall, the embedding suggests that the generator captures the structure of the GER spectral feature space but leaves a modest distributional gap that could be reduced with amplitude calibration or domain alignment.

#### 4.4.7. Fréchet Distance Analysis

**Figure 23:**
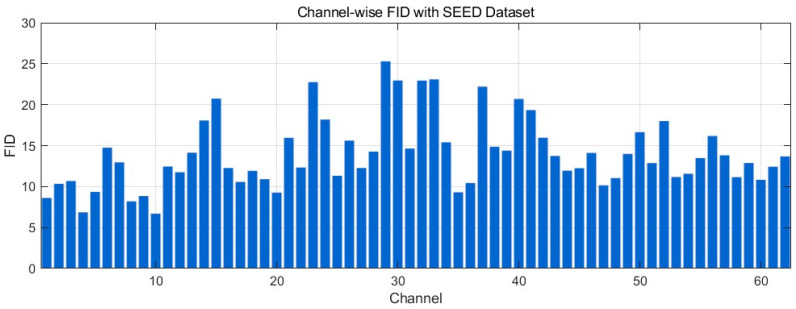
Channel-wise FID with SEED Dataset.

Figure 23 reports the channel-wise FID between real and generated EEG on the SEED dataset (lower is better). Most channels fall in a low–moderate range (~10–18), indicating broadly similar distributions of windowed spectral–temporal features. Several channels show elevated values (~20–26)—with a few local peaks near the mid-index channels—suggesting localized distributional gaps consistent with the small PSD/phase offsets observed in the time/frequency plots. Overall, the profile points to good distributional alignment for the majority of channels.

**Figure 24:**
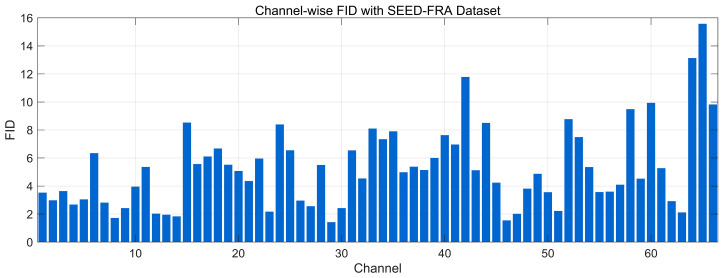
Channel-wise FID with SEED-FRA Dataset.

Figure 24 reports the channel-wise FID between real and generated EEG on the SEED-FRA dataset (lower is better). Most channels lie in a low range (~2–8), indicating close alignment of the windowed spectral–temporal feature distributions. Several channels show moderate elevations (~9–12), and a few late-index channels peak around ~13–16, suggesting localized distributional gaps consistent with the mild level/offset differences seen in the frequency-domain plots. Overall, the profile reflects good distributional similarity for the majority of channels.

**Figure 25:**
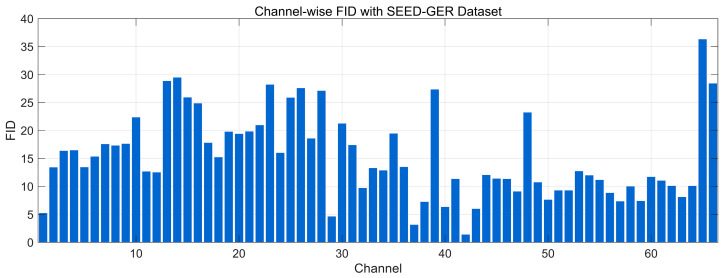
Channel-wise FID with SEED-GER Dataset.

Figure 25 reports the channel-wise FID between real and generated EEG on the SEED-GER dataset (lower is better). The majority of channels fall in a moderate range (~8–18), indicating broadly similar windowed spectral–temporal feature distributions. Several groups show elevated FID (~20–30), and there are isolated peaks near ~35–38 at late-index channels, suggesting localized distributional gaps consistent with the amplitude/offset differences and the centroid shift observed in the GER frequency and t-SNE analyses. A few channels reach low values (~2–6), showing near-match distributions. Overall, the profile points to good but less uniform alignment on SEED-GER than on SEED-FRA.

#### 4.4.8. MSE Analysis

**Figure 26:**
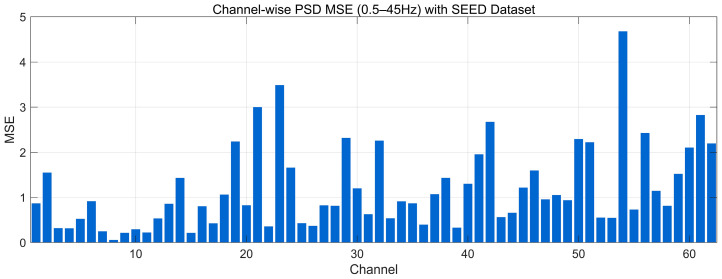
Channel-wise PSD MSE (0–45 Hz) with SEED Dataset.

Figure 26 reports the channel-wise PSD-MSE between the 0.5–45 Hz Welch spectra of real and generated EEG on the SEED dataset (lower is better). Most channels fall below ~1.0, indicating close agreement of spectral envelopes; a second band lies around ~1.0–2.0, and a few sparse peaks reach ~2.5–3.5, with an isolated maximum near ~4.8 on a late-index channel. These spikes suggest localized band-power mismatches rather than global distortion. Overall, the profile indicates broad spectral similarity across channels with a small subset showing larger deviations that align with the minor discrepancies observed in the PSD and KL/EMD analyses.

**Figure 27:**
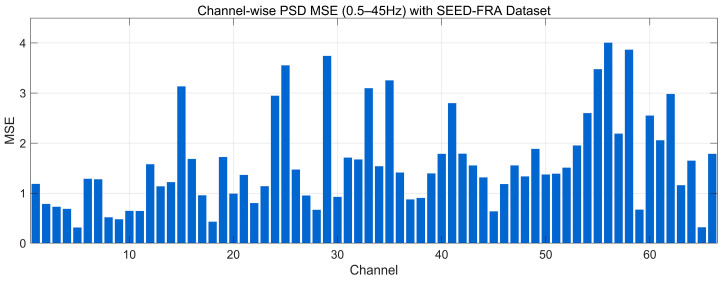
Channel-wise PSD MSE (0–45 Hz) with SEED-FRA Dataset.

Figure 27 reports the channel-wise PSD-MSE between the 0.5–45 Hz Welch spectra of real and generated EEG on the SEED-FRA dataset (lower is better). The bulk of channels cluster around ~0.6–1.6, indicating generally close spectral-envelope agreement; a secondary band appears at ~1.6–2.6, and several peaks rise to ~3.0–4.1 over late-index channels. These higher values suggest localized band-power mismatches rather than global distortions, consistent with the modest spectral offsets observed in the FRA PSD and the elevated EMD/KL on a subset of channels. Overall, the profile reflects good but less uniform frequency-domain alignment than SEED, with a small group of channels driving the larger errors.

**Figure 28:**
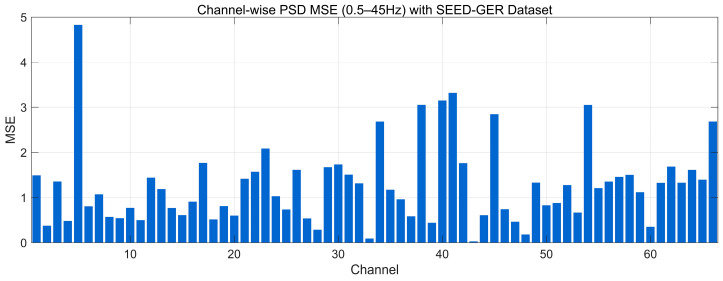
Channel-wise PSD MSE (0–45 Hz) with SEED-GER Dataset.

Figure 28 reports the channel-wise PSD-MSE between the 0.5–45 Hz Welch spectra of real and generated EEG on the SEED-GER dataset (lower is better). Most channels lie in a low range (~0.4–1.5), indicating broadly similar spectral envelopes; a secondary band appears around ~1.5–2.5, and several isolated peaks rise to ~3.0–3.4, with an early-index outlier near ~4.8. These spikes suggest localized band-power mismatches rather than global distortion, consistent with the modest offsets seen in the GER PSD and the higher FID/EMD on a small subset of channels. Overall, the profile indicates good but less uniform frequency-domain alignment on SEED-GER, with a few channels driving the larger errors.

#### 4.4.9. Earth Mover’s Distance Analysis

**Figure 29:**
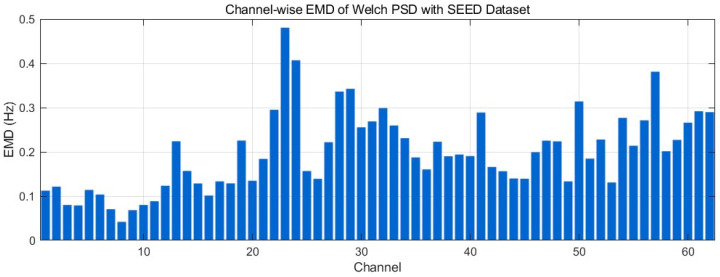
Channel-wise EMD of Welch PSD with SEED Dataset.

Figure 29 reports the channel-wise Earth Mover’s Distance (EMD) between the 0.5–45 Hz Welch PSDs of real and generated EEG on the SEED dataset (units: Hz, lower is better). Most channels lie in a low range (~0.08–0.20 Hz), indicating small frequency-wise mass shifts and broadly preserved spectral envelopes. A subset rises to ~0.20–0.30 Hz, and there are isolated peaks around ~0.35–0.45 Hz at mid/late-index channels, reflecting localized band-power redistributions rather than global mismatch. Overall, the profile suggests good frequency-domain alignment with a few channels exhibiting modest spectral shifts, consistent with the minor offsets seen in the PSD comparisons.

**Figure 30:**
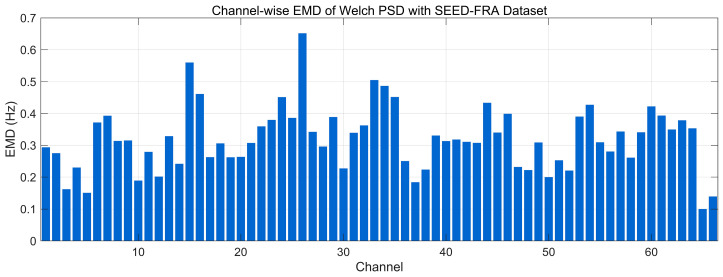
Channel-wise EMD of Welch PSD with SEED-FRA Dataset.

Figure 30 reports the channel-wise Earth Mover’s Distance (EMD) between the 0.5–45 Hz Welch PSDs of real and generated EEG on the SEED-FRA dataset (units: Hz, lower is better). Most channels fall in a moderate range (~0.20–0.40 Hz), indicating broadly preserved spectral envelopes with noticeable but bounded frequency-wise mass shifts. Several channels rise to ~0.45–0.55 Hz, and isolated peaks approach ~0.60–0.65 Hz (mid-index channels), reflecting localized band-power redistributions rather than global mismatch. Overall, the profile suggests good but less uniform frequency-domain alignment on SEED-FRA compared with SEED, consistent with cohort/device variability and the small spectral level offsets observed in the PSD comparisons.

**Figure 31:**
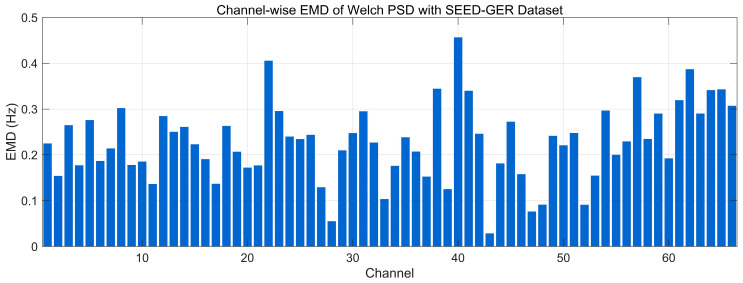
Channel-wise EMD of Welch PSD with SEED-GER Dataset.

Figure 31 reports the channel-wise Earth Mover’s Distance (EMD) between the 0.5–45 Hz Welch PSDs of real and generated EEG on the SEED-GER dataset (units: Hz, lower is better). Most channels lie in a low–moderate range (~0.12–0.30 Hz), indicating broadly preserved spectral envelopes. Several channels rise to ~0.30–0.38 Hz, and there are isolated peaks near ~0.40–0.47 Hz around mid- and late-index channels, reflecting localized band-power shifts rather than global mismatch. Overall, the profile suggests good frequency-domain alignment on SEED-GER with a small subset of channels exhibiting higher EMD, consistent with the modest offsets seen in the GER PSD and t-SNE analyses.

#### 4.4.10. Classification Consistency Analysis

Table 1 lists the mean ± SD across subjects. For completeness, this research includes AUC alongside Accuracy/Precision/Recall/F1. Following the protocol in Section 4.3.7, this research evaluates frozen-classifier congruence and augmentation utility on SEED, SEED-FRA, and SEED-GER.

In frozen-classifier congruence, a CNN trained only on real data is frozen and used to score generated segments. Across datasets, performance on generated data approaches but remains below the real-data baseline: accuracy differences are –0.046 (SEED: 0.823 vs. 0.869), –0.042 (SEED-FRA: 0.793 vs. 0.835), and –0.040 (SEED-GER: 0.809 vs. 0.849). Precision/Recall/F1 exhibits the same trend, indicating that synthesized signals are largely interpretable by a classifier trained on real data, while a modest domain gap persists.

For augmentation utility, when class-balanced generated segments are added to the real training set (validation/test kept real-only, hyper-parameters unchanged), mean performance consistently improves: accuracy gains are +0.049 (SEED: 0.918 vs. 0.869), +0.049 (SEED-FRA: 0.884 vs. 0.83the 5), and +0.045 (SEED-GER: 0.894 vs. 0.849), with concomitant improvements in F1 and AUC (Table 1, Table 2 and Table 3). These results indicate that the generator contributes non-redundant, label-consistent variability that enhances generalization rather than introducing noise.

### 4.5. Ablation Study

#### 4.5.1. Experimental Setup

To examine the contribution of each module and training strategy, this research conducts single-factor ablations in which one component (architecture, conditioning, or loss) is removed or replaced while all other configurations (optimizer, schedule, batch size, epochs, preprocessing, splits) are kept identical. The tested variants include: Without cVAE (remove KL, latent becomes deterministic), Without GAN (remove discriminator and adversarial loss), Without Label Conditioning (no conditional label embedding labels in encoder/decoder), Without Positional Embedding, Without Transformer Encoder (replace with a linear mapping), Transformer → CNN (1-D conv + pooling instead of attention), Without Pearson Loss, and the Baseline (full model with Transformer encoder, positional information, label conditioning, cVAE, GAN, Pearson + smoothness + spectral losses, and joint optimization).

#### 4.5.2. Results and Analysis

As shown in Table 4, the Baseline attains the best agreement (Pearson 0.838 ± 0.075, Spearman 0.819 ± 0.068) with comparatively low distributional gaps (KL 0.389 ± 0.145, FID 13.962 ± 4.293, EMD 0.198 ± 0.089) in the SEED dataset. Removing cVAE or GAN causes large drops in correlation (Pearson ≈ 0.51–0.52, Spearman ≈ 0.50–0.52) and markedly higher KL/FID/EMD (e.g., KL 1.476–1.545, FID 18.9–20.2, EMD 0.454–0.584), confirming the joint role of variational inference and adversarial learning in shaping a useful latent space and realistic samples. Label conditioning and positional information are also important: removing either degrades correlations to 0.61–0.63 and raises KL to 0.84–0.96. Eliminating the Transformer encoder further reduces Pearson/Spearman to 0.505/0.543. Replacing attention with a CNN partially recovers performance (Pearson 0.673 ± 0.145), but remains well below the Baseline, indicating the advantages of global attention over purely local convolution. Notably, ablation of the Pearson loss yields one of the worst distributional profiles (Pearson 0.454 ± 0.156, KL 1.735 ± 0.243, FID 21.445 ± 8.475, EMD 0.635 ± 0.234), underscoring its contribution to time-domain shape fidelity and its downstream effects on spectral/feature distributions.

As shown in Table 5, the Baseline reaches Pearson 0.739 ± 0.120 and Spearman 0.721 ± 0.117 with low FID (5.275 ± 2.906) relative to ablations in the SEED-FRA dataset. The most damaging removals are the Transformer encoder (Pearson 0.419 ± 0.201) and the adversarial/variational components (cVAE: 0.438, GAN: 0.427), all accompanied by strong increases in KL (1.54–2.15), FID (7.46–11.95), and EMD (0.65–0.79). Label conditioning and positional cues again provide clear gains (ablations at Pearson 0.516–0.579). CNN in place of attention yields 0.584 ± 0.175, above the linear mapping but below the Baseline. Pearson-loss removal (Pearson 0.554; KL 1.835) confirms that explicit trend alignment helps the broader objective mix on a cross-cohort corpus.

As shown in Table 6, the Baseline achieves the highest correlations (Pearson 0.844 ± 0.068, Spearman 0.831 ± 0.076) with modest KL (0.368 ± 0.184) in the SEED-GER dataset. Drops are evident when removing label conditioning (Pearson 0.437 ± 0.176) or Transformer (0.517 ± 0.157), and when ablating cVAE/GAN (Pearson 0.546–0.575). These degradations coincide with larger FID (17.3–19.6) and EMD (0.587–0.721), reflecting distributional shifts seen in GER’s t-SNE/PSD analyses. CNN replacement again performs mid-pack (Pearson 0.681 ± 0.167), highlighting that global attention is beneficial even under device/cohort shifts. Removing the Pearson loss (Pearson 0.538, KL 0.735) still harms overall alignment, though its effect size is somewhat smaller than on SEED/FRA.

Across all three datasets, the full model consistently yields the best correlation metrics and the lowest KL/FID/EMD. The largest degradations arise from removing cVAE/GAN or the Transformer; label conditioning and positional information give non-trivial gains; CNN recovers part of the attention benefits but not all. The Pearson loss materially improves time-domain morphology and, indirectly, frequency-domain and distributional alignment. These patterns support the necessity and complementarity of the proposed architectural and objective choices.

### 4.6. Comparative Experiments

#### 4.6.1. Experimental Setup

To establish a controlled comparison in terms of time–frequency fidelity and downstream usability, this research evaluates four representative GAN baselines—DCGAN, WGAN, WGAN-GP, and T-CGAN [32]—under identical data, preprocessing, and training protocols. T-CGAN conditions both the generator and discriminator on time stamps to model irregularly sampled time series. All models are trained and assessed on the SEED, SEED-FRA, and SEED-GER datasets with the same within-subject, trial-level split. Evaluation comprises channel-wise Pearson and Spearman correlations and distributional distances. Downstream consistency and augmentation value are examined with a unified CNN classifier using three regimes: Real-only (train/validation/test on real data), Generated-only → Real test (train on generated, validate/test on real), and Real + Generated (augment the real training set with class-balanced generated segments while keeping validation/test real-only).

#### 4.6.2. Results and Analysis

The results of comparative experiments were summarized in Table 7, Table 8, Table 9, Table 10, Table 11 and Table 12. Across datasets, the proposed baseline achieves the highest similarity and the lowest distributional discrepancy. On SEED (Table 7), the baseline records Pearson 0.838 ± 0.075, Spearman 0.819 ± 0.068, KL 0.389 ± 0.145, FID 13.962 ± 4.293, and EMD 0.198 ± 0.089, outperforming the GAN baselines; among them, WGAN-GP is consistently the strongest competitor (e.g., Pearson 0.629 ± 0.084, Spearman 0.694 ± 0.121, KL 0.459 ± 0.139), whereas DCGAN, WGAN, and T-CGAN show larger gaps in both correlation and spectral distance. The same pattern is observed on SEED-FRA (Table 9), where the baseline yields Pearson 0.739 ± 0.120, Spearman 0.721 ± 0.117, KL 0.411 ± 0.195, FID 5.275 ± 2.906, and EMD 0.320 ± 0.100, with WGAN-GP again ranking second among baselines. On SEED-GER (Table 11), the baseline remains best (Pearson 0.844 ± 0.068, Spearman 0.831 ± 0.076, KL 0.368 ± 0.184, FID 15.308 ± 7.523, EMD 0.227 ± 0.084), indicating robustness under cohort/device shifts, while WGAN-GP is again the closest challenger, and the other baselines trail further behind.

Downstream classification corroborates these consistency results. Under the Generated-only → Real test protocol (Table 8, Table 10 and Table 12), performance remains below the Real-only baseline for all generators, reflecting a domain gap when the classifier has never seen real data. Nevertheless, the baseline generator attains the strongest transfer among competitors on each dataset (SEED: 0.823 ± 0.186; SEED-FRA: 0.793 ± 0.176; SEED-GER: 0.809 ± 0.204 in accuracy). When generated segments are used for training set augmentation with validation/test kept real-only, the baseline achieves the largest and most consistent gains—SEED: 0.918 ± 0.094 vs. 0.869 ± 0.102; SEED-FRA: 0.884 ± 0.089 vs. 0.835 ± 0.112; SEED-GER: 0.894 ± 0.102 vs. 0.849 ± 0.113—accompanied by concomitant improvements in F1 and AUC. These outcomes indicate that the baseline not only preserves time-domain morphology and spectral envelopes more faithfully (high correlation, low KL/FID/EMD) but also contributes label-consistent, non-redundant variability that enhances classifier generalization. Overall, merely altering the adversarial loss (DCGAN, WGAN, WGAN-GP, T-CGAN) is insufficient to match the balanced, cross-dataset performance achieved by the proposed baseline, which integrates variational modeling, conditional control, and attention-based sequence representation.

## 5. Discussion

### 5.1. Challenges of EEG Signal Structural Characteristics for Generative Modeling

EEG’s non-stationarity, multi-scale rhythms, and cross-channel coupling make faithful generation difficult, and the cross-dataset ablations (SEED/SEED-FRA/SEED-GER) consistently reflect this. The Baseline yields the strongest balance of time-domain similarity and spectral fidelity (e.g., SEED Pearson/Spearman ≈ 0.84/0.82, KL ≈ 0.39), while removing cVAE or GAN markedly lowers correlations (Pearson ≈ 0.43–0.58) and inflates KL (≈0.75–1.68). Dropping label conditioning further degrades both temporal and spectral metrics, confirming its role in emotion-specific structure. Eliminating positional encoding or the Transformer encoder weakens long-range temporal modeling and raises spectral errors (KL often >0.9, up to ~2.15 on SEED-FRA). Removing the Pearson loss uniformly worsens morphology and spectrum (higher KL/FID/EMD). Taken together, variational modeling, adversarial refinement, label conditioning, positional encoding, and attention are all necessary to counter EEG’s intrinsic dynamics; the full model achieves the most stable trade-off across datasets.

### 5.2. Applicability and Complementarity of Multi-Dimensional Evaluation Metrics

A multi-metric perspective proves essential for EEG generation, as time-domain similarity and frequency-domain fidelity do not always covary. Across SEED, the Baseline achieves the most balanced profile—high temporal agreement (Pearson 0.838 ± 0.075, Spearman 0.819 ± 0.068) together with low spectral divergence (KL 0.389 ± 0.145, EMD 0.198 ± 0.089) and competitive FID (13.962 ± 4.293)—whereas classical GAN variants (DCGAN, WGAN, WGAN-GP, T-CGAN) exhibit mixed behavior: some improve one axis slightly (e.g., WGAN-GP modestly better Spearman 0.694 ± 0.121 than other baselines) yet incur higher KL/EMD (e.g., KL 0.459–0.635, EMD 0.257–0.424) or lower correlations (Pearson typically 0.54–0.63). The same pattern holds on SEED-FRA and SEED-GER, where the Baseline simultaneously maintains stronger correlations and lower KL/EMD than competing GANs. Importantly, downstream classification aligns with these multi-metric trends: under a frozen real-trained CNN, generated segments approach real-data performance (e.g., SEED accuracy 0.823 ± 0.186 vs. 0.869 ± 0.102), and augmenting real training sets with generated segments consistently improves validation/test results while keeping evaluation real-only (SEED 0.918 ± 0.094, SEED-FRA 0.884 ± 0.089, SEED-GER 0.894 ± 0.102 accuracy). These findings indicate that single metrics are insufficient to certify signal quality; rather, concordance across complementary measures—temporal correlations, spectral divergence (KL/EMD), and FID—together with downstream gains provides a robust assessment, and the Baseline’s balanced superiority across these axes supports its practical effectiveness.

### 5.3. Challenges in the Interpretability of Generative Models

Despite these improvements, generative modeling for EEG remains hindered by interpretability challenges. The Baseline model outperforms conventional GAN variants in both temporal and spectral domains, yet the internal mechanisms that link latent representations to neurophysiological features remain opaque. Like most deep generative models, the framework functions as a “black box”, limiting its clinical trustworthiness and controllability. Future research should, therefore, emphasize interpretability-oriented strategies, such as incorporating guided latent variable modeling, integrating neurophysiological priorities, or applying post hoc tools such as SHAP and LIME to map latent dimensions to cognitive or emotional processes. Enhancing interpretability will not only improve mechanistic understanding but also facilitate reliable applications in clinical diagnosis, neuroregulation, and brain–computer interfaces.

### 5.4. Loss Design Under Non-Stationary EEG

EEG dynamics are non-stationary, so time-domain objectives that admit free lags or warping, such as cross-correlation with non-zero lags, may reward temporal shifts rather than reproducing morphology, which is misaligned with our goal of phase-aware waveform fidelity. This research, therefore, pairs a zero-lag, mean/variance-normalized Pearson term, which enforces shape and phase alignment, with a Welch-PSD term, which constrains the spectral envelope. In the ablation experiment, removing the Pearson term produced the largest drop in time-domain fidelity, while the spectrum loss remained complementary. More expressive objectives, such as coherence or phase-locking value measures and time–frequency losses via STFT or CWT, could be explored, but they introduce additional hyperparameters and computation; this research leaves these extensions for future work.

### 5.5. Practical Fidelity for Classification

While several similarity metrics exhibit moderate averages (e.g., channel-wise Pearson/Spearman and band-agnostic PSD errors), these indicators are conservative for short, non-stationary EEG windows. What matters for application is whether the synthesized signals preserve label-relevant structure in aggregate across channels and frequencies. This is corroborated by the frozen-classifier congruence and augmentation analyses (Section 4.4.10; Table 1, Table 2 and Table 3): generated segments scored by a CNN trained on real data approach the real-data baseline, and adding generated data to the real training set consistently improves mean accuracy/precision/recall/F1 (validation/test kept real-only), indicating non-redundant, label-consistent variability rather than noise.

## 6. Limitations

The evaluation is restricted to the SEED family SEED, SEED-FRA, and SEED-GER and does not include heterogeneous datasets such as DEAP or DREAMER with different montages, sampling rates, referencing schemes, trial structures, and continuous labels. This research also did not assess cross-device robustness or cross-device generation meaningful extension will require channel harmonization, label-space alignment, and device-aware adaptation via normalization, re-referencing, and domain alignment. Architecture ablations are removal-style and reported as diagnostic under inter-module dependencies, whereas loss ablations are interface-preserving. A full replacement, interface-preserving protocol with identity/linear adapters and parameter-matched substitutes is left for future work to further isolate causal effects. Moreover, this research does not quantify conditional controllability. In particular, fixed-latent label-swap experiments and the associated label-consistency metrics are left for future work.

## 7. Conclusions

This study proposed a Trans-cVAE-GAN framework for EEG signal generation, which integrates label conditioning, adversarial training, and variational inference to address the challenges of non-stationarity and spatiotemporal coupling in EEG data. Experimental results demonstrated that the model achieves superior performance over conventional GAN variants, with ablation studies confirming the necessity of each key component. The generated signals not only preserved temporal and spectral consistency but also enhanced downstream emotion classification, highlighting the model’s potential for EEG data augmentation and brain–computer interface applications.

## Figures and Tables

**Table 1:** Classification Consistency Analysis with SEED Dataset.

Data Regime	Train Set	Validation/Test	Accuracy (Mean ± SD)	Precision (Mean ± SD)	Recall (Mean ± SD)	F1-Score (Mean ± SD)	AUC (Mean ± SD)
Real EEG	Real	Real	0.869 ± 0.102	0.863 ± 0.114	0.859 ± 0.132	0.860 ± 0.126	0.857 ± 0.107
Generated EEG	Generated	Real	0.823 ± 0.186	0.831 ± 0.194	0.834 ± 0.176	0.836 ± 0.182	0.832 ± 0.190
Real + Generated EEG	Real + Generated	Real	0.918 ± 0.094	0.921 ± 0.087	0.918 ± 0.096	0.903 ± 0.077	0.917 ± 0.084

**Table 2:** Classification Consistency Analysis with SEED-FRA Dataset.

Data Regime	Train Set	Validation/Test	Accuracy (Mean ± SD)	Precision (Mean ± SD)	Recall (Mean ± SD)	F1-Score (Mean ± SD)	AUC (Mean ± SD)
Real EEG	Real	Real	0.835 ± 0.112	0.831 ± 0.121	0.839 ± 0.117	0.836 ± 0.124	0.826 ± 0.124
Generated EEG	Generated	Real	0.793 ± 0.176	0.784 ± 0.176	0.795 ± 0.169	0.782 ± 0.180	0.787 ± 0.173
Real + Generated EEG	Real + Generated	Real	0.884 ± 0.089	0.881 ± 0.073	0.878 ± 0.082	0.876 ± 0.074	0.881 ± 0.081

**Table 3:** Classification Consistency Analysis with SEED-GER Dataset.

Data Regime	Train Set	Validation/Test	Accuracy (Mean ± SD)	Precision (Mean ± SD)	Recall (Mean ± SD)	F1-Score (Mean ± SD)	AUC (Mean ± SD)
Real EEG	Real	Real	0.849 ± 0.113	0.854 ± 0.115	0.846 ± 0.121	0.853 ± 0.117	0.853 ± 0.119
Generated EEG	Generated	Real	0.809 ± 0.204	0.812 ± 0.210	0.808 ± 0.198	0.812 ± 0.202	0.814 ± 0.213
Real + Generated EEG	Real + Generated	Real	0.894 ± 0.102	0.892 ± 0.107	0.901 ± 0.113	0.903 ± 0.097	0.896 ± 0.104

**Table 4:** Ablation Study Results with SEED Dataset.

Model	Pearson (Mean ± SD)	Spearman (Mean ± SD)	KL Divergence (Mean ± SD)	FID (Mean ± SD)	EMD (Mean ± SD)
Baseline	0.838 ± 0.075	0.819 ± 0.068	0.389 ± 0.145	13.962 ± 4.293	0.198 ± 0.089
Without cVAE	0.512 ± 0.104	0.516 ± 0.122	1.476 ± 0.254	18.894 ± 8.058	0.454 ± 0.243
Without GAN	0.521 ± 0.131	0.504 ± 0.132	1.545 ± 0.247	20.156 ± 8.156	0.584 ± 0.215
Without Label Conditioning	0.634 ± 0.086	0.621 ± 0.107	0.843 ± 0.176	16.453 ± 7.534	0.345 ± 0.156
Without Positional Embedding	0.612 ± 0.073	0.635 ± 0.096	0.957 ± 0.169	15.346 ± 6.453	0.376 ± 0.175
Without Pearson Loss	0.454 ± 0.156	0.476 ± 0.185	1.735 ± 0.243	21.445 ± 8.475	0.635 ± 0.234
Without Transformer Encoder	0.505 ± 0.116	0.543 ± 0.121	1.246 ± 0.028	19.473 ± 8.456	0.548 ± 0.164
Transformer → CNN	0.673 ± 0.145	0.667 ± 0.175	1.333 ± 0.168	18.437 ± 8.045	0.534 ± 0.237

**Table 5:** Ablation Study Results with SEED-FRA Dataset.

Model	Pearson (Mean ± SD)	Spearman (Mean ± SD)	KL Divergence (Mean ± SD)	FID (Mean ± SD)	EMD (Mean ± SD)
Baseline	0.739 ± 0.120	0.721 ± 0.117	0.411 ± 0.195	5.275 ± 2.906	0.320 ± 0.100
Without cVAE	0.438 ± 0.183	0.413 ± 0.197	1.537 ± 0.354	9.453 ± 5.234	0.731 ± 0.315
Without GAN	0.427 ± 0.197	0.454 ± 0.204	1.678 ± 0.423	10.049 ± 4.456	0.794 ± 0.434
Without Label Conditioning	0.579 ± 0.164	0.543 ± 0.172	1.435 ± 0.275	8.134 ± 4.435	0.683 ± 0.286
Without Positional Embedding	0.516 ± 0.157	0.523 ± 0.184	1.535 ± 0.434	7.464 ± 3.537	0.647 ± 0.307
Without Pearson Loss	0.554 ± 0.146	0.549 ± 0.135	1.835 ± 0.354	7.587 ± 4.241	0.681 ± 0.314
Without Transformer Encoder	0.419 ± 0.201	0.425 ± 0.201	2.154 ± 0.546	11.946 ± 6.028	0.754 ± 0.412
Transformer → CNN	0.584 ± 0.175	0.548 ± 0.186	1.945 ± 0.712	7.944 ± 5.453	0.657 ± 0.284

**Table 6:** Ablation Study Results with SEED-GER Dataset.

Model	Pearson (Mean ± SD)	Spearman (Mean ± SD)	KL Divergence (Mean ± SD)	FID (Mean ± SD)	EMD (Mean ± SD)
Baseline	0.844 ± 0.068	0.831 ± 0.076	0.368 ± 0.184	15.308 ± 7.523	0.227 ± 0.084
Without cVAE	0.575 ± 0.172	0.548 ± 0.134	0.745 ± 0.542	18.453 ± 6.457	0.646 ± 0.143
Without GAN	0.546 ± 0.195	0.512 ± 0.143	0.764 ± 0.459	19.378 ± 8.435	0.682 ± 0.187
Without Label Conditioning	0.437 ± 0.176	0.487 ± 0.154	0.845 ± 0.453	19.547 ± 7.945	0.721 ± 0.195
Without Positional Embedding	0.543 ± 0.201	0.537 ± 0.194	0.794 ± 0.494	18.647 ± 7.547	0.675 ± 0.157
Without Pearson Loss	0.538 ± 0.168	0.546 ± 0.168	0.735 ± 0.427	17.287 ± 7.684	0.587 ± 0.135
Without Transformer Encoder	0.517 ± 0.157	0.508 ± 0.121	0.935 ± 0.543	17.548 ± 8.054	0.594 ± 0.154
Transformer → CNN	0.681 ± 0.167	0.654 ± 0.135	0.673 ± 0.354	18.387 ± 8.154	0.543 ± 0.123

**Table 7:** Comparative Experiment Statistical Results with SEED Dataset.

Model	Pearson (Mean ± SD)	Spearman (Mean ± SD)	KL Divergence (Mean ± SD)	FID (Mean ± SD)	EMD (Mean ± SD)
Baseline	0.838 ± 0.075	0.819 ± 0.068	0.389 ± 0.145	13.962 ± 4.293	0.198 ± 0.089
DCGAN	0.543 ± 0.096	0.576 ± 0.105	0.635 ± 0.234	15.436 ± 3.957	0.323 ± 0.153
WGAN	0.567 ± 0.126	0.586 ± 0.135	0.536 ± 0.142	14.954 ± 4.982	0.424 ± 0.183
WGAN-GP	0.629 ± 0.084	0.694 ± 0.121	0.459 ± 0.139	14.532 ± 4.531	0.257 ± 0.136
T-CGAN	0.624 ± 0.093	0.657 ± 0.119	0.546 ± 0.176	15.168 ± 4.587	0.275 ± 0.149

**Table 8:** Comparative Experiment Classification Results with SEED Dataset.

Model	Train Set	Validation/Test	Accuracy (Mean ± SD)	Precision (Mean ± SD)	Recall (Mean ± SD)	F1-Score (Mean ± SD)	AUC (Mean ± SD)
Baseline	Real	Real	0.869 ± 0.102	0.863 ± 0.114	0.859 ± 0.132	0.860 ± 0.126	0.857 ± 0.107
	Generated	Real	0.823 ± 0.186	0.831 ± 0.194	0.834 ± 0.176	0.836 ± 0.182	0.832 ± 0.190
	Real + Generated	Real	0.918 ± 0.094	0.921 ± 0.087	0.918 ± 0.096	0.903 ± 0.077	0.917 ± 0.084
DCGAN	Real	Real	0.869 ± 0.102	0.863 ± 0.114	0.859 ± 0.132	0.860 ± 0.126	0.857 ± 0.107
	Generated	Real	0.803 ± 0.167	0.809 ± 0.186	0.806 ± 0.172	0.798 ± 0.163	0.796 ± 0.171
	Real + Generated	Real	0.881 ± 0.096	0.878 ± 0.102	0.876 ± 0.109	0.874 ± 0.104	0.877 ± 0.098
WGAN	Real	Real	0.869 ± 0.102	0.863 ± 0.114	0.859 ± 0.132	0.860 ± 0.126	0.857 ± 0.107
	Generated	Real	0.817 ± 0.169	0.821 ± 0.173	0.819 ± 0.176	0.820 ± 0.167	0.823 ± 0.162
	Real + Generated	Real	0.891 ± 0.104	0.889 ± 0.106	0.893 ± 0.097	0.892 ± 0.101	0.891 ± 0.106
WGAN-GP	Real	Real	0.869 ± 0.102	0.863 ± 0.114	0.859 ± 0.132	0.860 ± 0.126	0.857 ± 0.107
	Generated	Real	0.821 ± 0.186	0.818 ± 0.176	0.823 ± 0.168	0.819 ± 0.173	0.820 ± 0.157
	Real + Generated	Real	0.904 ± 0.099	0.901 ± 0.103	0.897 ± 0.101	0.903 ± 0.096	0.899 ± 0.093
T-CGAN	Real	Real	0.869 ± 0.102	0.863 ± 0.114	0.859 ± 0.132	0.860 ± 0.126	0.857 ± 0.107
	Generated	Real	0.812 ± 0.168	0.807 ± 0.159	0.809 ± 0.172	0.813 ± 0.163	0.811 ± 0.159
	Real + Generated	Real	0.886 ± 0.102	0.883 ± 0.106	0.878 ± 0.096	0.886 ± 0.093	0.879 ± 0.104

**Table 9:** Comparative Experiment Statistical Results with SEED-FRA Dataset (mean ± sd).

Model	Pearson (Mean ± SD)	Spearman (Mean ± SD)	KL Divergence (Mean ± SD)	FID (Mean ± SD)	EMD (Mean ± SD)
Baseline	0.739 ± 0.120	0.721 ± 0.117	0.411 ± 0.195	5.275 ± 2.906	0.320 ± 0.100
DCGAN	0.496 ± 0.103	0.481 ± 0.138	0.589 ± 0.261	8.354 ± 3.984	0.631 ± 0.203
WGAN	0.547 ± 0.135	0.537 ± 0.186	0.573 ± 0.234	7.545 ± 3.533	0.538 ± 0.251
WGAN-GP	0.603 ± 0.129	0.684 ± 0.139	0.510 ± 0.211	7.371 ± 2.574	0.357 ± 0.086
T-CGAN	0.594 ± 0.096	0.583 ± 0.126	0.476 ± 0.186	8.163 ± 3.896	0.376 ± 0.168

**Table 10:** Comparative Experiment Classification Results with SEED-FRA Dataset.

Model	Train Set	Validation/Test	Accuracy (Mean ± SD)	Precision (Mean ± SD)	Recall (Mean ± SD)	F1-Score (Mean ± SD)	AUC (Mean ± SD)
Baseline	Real	Real	0.835 ± 0.112	0.831 ± 0.121	0.839 ± 0.117	0.836 ± 0.124	0.826 ± 0.124
	Generated	Real	0.793 ± 0.176	0.784 ± 0.176	0.795 ± 0.169	0.782 ± 0.180	0.787 ± 0.173
	Real + Generated	Real	0.884 ± 0.089	0.881 ± 0.073	0.878 ± 0.082	0.876 ± 0.074	0.881 ± 0.081
DCGAN	Real	Real	0.835 ± 0.112	0.831 ± 0.121	0.839 ± 0.117	0.836 ± 0.124	0.826 ± 0.124
	Generated	Real	0.773 ± 0.201	0.770 ± 0.197	0.768 ± 0.194	0.776 ± 0.193	0.774 ± 0.213
	Real + Generated	Real	0.851 ± 0.124	0.853 ± 0.138	0.849 ± 0.132	0.852 ± 0.135	0.847 ± 0.129
WGAN	Real	Real	0.835 ± 0.112	0.831 ± 0.121	0.839 ± 0.117	0.836 ± 0.124	0.826 ± 0.124
	Generated	Real	0.776 ± 0.186	0.779 ± 0.173	0.784 ± 0.174	0.777 ± 0.182	0.782 ± 0.179
	Real + Generated	Real	0.861 ± 0.116	0.863 ± 0.106	0.859 ± 0.124	0.868 ± 0.097	0.864 ± 0.107
WGAN-GP	Real	Real	0.835 ± 0.112	0.831 ± 0.121	0.839 ± 0.117	0.836 ± 0.124	0.826 ± 0.124
	Generated	Real	0.781 ± 0.163	0.776 ± 0.159	0.778 ± 0.168	0.783 ± 0.135	0.778 ± 0.143
	Real + Generated	Real	0.867 ± 0.102	0.871 ± 0.093	0.873 ± 0.086	0.869 ± 0.106	0.870 ± 0.112
T-CGAN	Real	Real	0.835 ± 0.112	0.831 ± 0.121	0.839 ± 0.117	0.836 ± 0.124	0.826 ± 0.124
	Generated	Real	0.773 ± 0.172	0.771 ± 0.176	0.768 ± 0.196	0.762 ± 0.189	0.776 ± 0.168
	Real + Generated	Real	0.849 ± 0.084	0.847 ± 0.086	0.851 ± 0.076	0.849 ± 0.081	0.850 ± 0.093

**Table 11:** Comparative Experiment Statistical Results with SEED-GER Dataset.

Model	Pearson (Mean ± SD)	Spearman (Mean ± SD)	KL Divergence (Mean ± SD)	FID (Mean ± SD)	EMD (Mean ± SD)
Baseline	0.844 ± 0.068	0.831 ± 0.076	0.368 ± 0.184	15.308 ± 7.523	0.227 ± 0.084
DCGAN	0.568 ± 0.168	0.594 ± 0.172	0.531 ± 0.306	18.354 ± 8.461	0.513 ± 0.197
WGAN	0.506 ± 0.206	0.524 ± 0.234	0.608 ± 0.259	18.891 ± 8.648	0.672 ± 0.216
WGAN-GP	0.623 ± 0.106	0.648 ± 0.269	0.514 ± 0.183	17.541 ± 6.984	0.435 ± 0.105
T-CGAN	0.618 ± 0.083	0.615 ± 0.091	0.481 ± 0.241	17.764 ± 7.948	0.437 ± 0.117

**Table 12:** Comparative Experiment Classification Results with SEED-GER Dataset.

Model	Train Set	Validation/Test	Accuracy (Mean ± SD)	Precision (Mean ± SD)	Recall (Mean ± SD)	F1-Score (Mean ± SD)	AUC (Mean ± SD)
Baseline	Real	Real	0.849 ± 0.113	0.854 ± 0.115	0.846 ± 0.121	0.853 ± 0.117	0.853 ± 0.119
	Generated	Real	0.809 ± 0.204	0.812 ± 0.210	0.808 ± 0.198	0.812 ± 0.202	0.814 ± 0.213
	Real + Generated	Real	0.894 ± 0.102	0.892 ± 0.107	0.901 ± 0.113	0.903 ± 0.097	0.896 ± 0.104
DCGAN	Real	Real	0.849 ± 0.113	0.854 ± 0.115	0.846 ± 0.121	0.853 ± 0.117	0.853 ± 0.119
	Generated	Real	0.768 ± 0.189	0.772 ± 0.176	0.767 ± 0.184	0.756 ± 0.167	0.761 ± 0.172
	Real + Generated	Real	0.867 ± 0.119	0.863 ± 0.123	0.861 ± 0.117	0.867 ± 0.120	0.862 ± 0.131
WGAN	Real	Real	0.849 ± 0.113	0.854 ± 0.115	0.846 ± 0.121	0.853 ± 0.117	0.853 ± 0.119
	Generated	Real	0.773 ± 0.173	0.772 ± 0.154	0.768 ± 0.168	0.764 ± 0.171	0.774 ± 0.169
	Real + Generated	Real	0.872 ± 0.083	0.869 ± 0.094	0.873 ± 0.102	0.864 ± 0.099	0.871 ± 0.082
WGAN-GP	Real	Real	0.849 ± 0.113	0.854 ± 0.115	0.846 ± 0.121	0.853 ± 0.117	0.853 ± 0.119
	Generated	Real	0.791 ± 0.110	0.793 ± 0.103	0.801 ± 0.109	0.796 ± 0.104	0.794 ± 0.096
	Real + Generated	Real	0.883 ± 0.106	0.886 ± 0.092	0.879 ± 0.109	0.881 ± 0.086	0.884 ± 0.093
T-CGAN	Real	Real	0.849 ± 0.113	0.854 ± 0.115	0.846 ± 0.121	0.853 ± 0.117	0.853 ± 0.119
	Generated	Real	0.763 ± 0.194	0.772 ± 0.183	0.768 ± 0.173	0.765 ± 0.169	0.772 ± 0.164
	Real + Generated	Real	0.863 ± 0.112	0.861 ± 0.094	0.869 ± 0.106	0.871 ± 0.109	0.873 ± 0.097

## Data Availability

No new data were created or analyzed in this study. Data sharing is not applicable to this article. The data presented in this study are available in Shanghai Jiaotong University at 10.1109/TAMD.2015.2431497, 10.1109/NER.2013.6695876, 10.1088/1741-2552/ac5c8d. These data were derived from the following resources available in the public domain: https://bcmi.sjtu.edu.cn/home/seed/seed.html, https://bcmi.sjtu.edu.cn/home/seed/seed-FRA.html, https://bcmi.sjtu.edu.cn/home/seed/seed-GER.html (all accessed on 23 September 2025).
